# A compression algorithm for the combination of PDF sets

**DOI:** 10.1140/epjc/s10052-015-3703-3

**Published:** 2015-10-05

**Authors:** Stefano Carrazza, José I. Latorre, Juan Rojo, Graeme Watt

**Affiliations:** Dipartimento di Fisica, Università di Milano and INFN, Sezione di Milano, Via Celoria 16, 20133 Milan, Italy; Departament d’Estructura i Constituents de la Matèria, Universitat de Barcelona, Diagonal 647, 08028 Barcelona, Spain; Rudolf Peierls Centre for Theoretical Physics, University of Oxford, 1 Keble Road, Oxford, OX1 3NP UK; Institute for Particle Physics Phenomenology, Durham University, Durham, DH1 3LE UK

## Abstract

The current PDF4LHC recommendation to estimate uncertainties due to parton distribution functions (PDFs) in theoretical predictions for LHC processes involves the combination of separate predictions computed using PDF sets from different groups, each of which comprises a relatively large number of either Hessian eigenvectors or Monte Carlo (MC) replicas. While many fixed-order and parton shower programs allow the evaluation of PDF uncertainties for a single PDF set at no additional CPU cost, this feature is not universal, and, moreover, the a posteriori combination of the predictions using at least three different PDF sets is still required. In this work, we present a strategy for the statistical combination of individual PDF sets, based on the MC representation of Hessian sets, followed by a compression algorithm for the reduction of the number of MC replicas. We illustrate our strategy with the combination and compression of the recent NNPDF3.0, CT14 and MMHT14 NNLO PDF sets. The resulting compressed Monte Carlo PDF sets are validated at the level of parton luminosities and LHC inclusive cross sections and differential distributions. We determine that around 100 replicas provide an adequate representation of the probability distribution for the original combined PDF set, suitable for general applications to LHC phenomenology.

## Introduction

Parton distribution functions (PDFs) are an essential ingredient for LHC phenomenology [1–7]. They are one of the limiting theory factors for the extraction of Higgs couplings from LHC data [8], they reduce the reach of many BSM searches, particularly in the high-mass region [9–11], and they are the dominant source of systematic uncertainty in precision electroweak measurements such as the *W* mass at the LHC [12–14]. A crucial question is therefore how to estimate the total PDF uncertainty that affects the various processes listed above.

While modern PDF sets [15–22] provide their own estimates of the associated PDF error, using a single set might not lead to a robust enough estimate of the total uncertainty arising from our imperfect knowledge of the PDFs in LHC computations. For instance, different global PDF sets, based on similar input datasets and theory assumptions, while in reasonable agreement, can still differ for some PDF flavors and $(x,Q^2)$ regions by a non-negligible amount [4, 5, 23]. These differences are likely to arise from the different fitting methodologies or from sources of theoretical uncertainty that are not yet accounted for, such as missing higher orders or parametric uncertainties. For these reasons, while an improved understanding of the origin of these differences is achieved, from the practical point of view it is necessary to combine different PDF sets to obtain a more reliable estimate of the total PDF uncertainty in LHC applications.

That was the motivation underlying the original 2010 recommendation from the PDF4LHC Working Group to compute the total PDF uncertainty in LHC processes [24, 25]. The prescription was to take the envelope and midpoint of the three global sets available at the time (CTEQ6.6 [26], MSTW08 [27] and NNPDF2.0 [28]), each at their default value of $\alpha _s(M_Z)$, and where each set included the combined PDF+$\alpha _s$ uncertainty using the corresponding prescription [29–31]. This prescription has been updated [32] to the most recent sets from each group, and currently these are CT14 [22], MMHT14 [19] and NNPDF3.0 [16]. More recently, PDF4LHC has simplified the prescription for the combination of PDF+$\alpha _s$ uncertainties: the current recommendation [33] is now to take the three global sets at a common value of $\alpha _s(M_Z)=0.118$, close enough to the most recent PDG average [34], and then add in quadrature the additional uncertainty due to $\alpha _s$. This procedure has been shown to be exact within the Gaussian approximation in the case of CT [29], and close enough to the exact prescription for practical applications in the cases of MMHT and NNPDF [30, 31].

One criticism that has been raised to this PDF4LHC recommendation is that defining the total PDF uncertainty by the envelope of the predictions from different sets does not have a well-defined statistical interpretation. However, as originally proposed by Forte in [1], and developed in some more detail later by Forte and Watt in [2, 35, 36], it is possible to modify the PDF4LHC prescription to give the combination of PDF sets a robust statistical meaning as follows. The first step consists in transforming the Hessian PDF sets into Monte Carlo (MC) PDF sets using the Watt and Thorne method [35]. Then one can consider that each of the replicas from each set is a different instance of a common probability distribution, thus the combination of the different sets can be achieved by simply adding together their Monte Carlo replicas. Assuming that each PDF set that enters the combination has the same a priori probability, the same number of replicas should be chosen from each set. The predictions from this combined Monte Carlo PDF set, which now clearly have a well-defined statistical meaning, turn out to be in reasonable agreement from those of the original envelope and midpoint method proposed by PDF4LHC. However, the resulting PDF uncertainties will generally be slightly smaller, since the envelope method gives more weight to the outliers than the MC combination method.

In general, any method for the combination of PDF sets from different groups presents practical difficulties at the implementation level. The first one is purely computational: theoretical predictions have to be computed from all the eigenvectors/replicas of the various PDF sets, which in total require the same calculation to be redone around $\mathcal {O}\left( 200\right)$ times for the PDF4LHC envelope or around $\mathcal {O}\left( 900\right)$ times for the Monte Carlo combination, a very CPU-intensive task. Fortunately, some of the most widely used Monte Carlo event generators, such as MadGraph5_aMC@NLO [37, 38] or POWHEG [39], and NNLO codes like FEWZ [40], now allow computation of PDF uncertainties at no extra cost. However, this is not the case for all the theory tools used for the LHC experiments, and even when this feature is available, in the case of the envelope method the a posteriori combination of the results obtained with the three sets still needs to be performed, which can be quite cumbersome (as well as error-prone) especially in the case of exclusive calculations that require very large event files.

The above discussion provides the motivation to develop new strategies for the combination of individual PDF sets, and the subsequent reduction to a small number of eigenvectors or replicas. One possible approach in this direction, the Meta-PDFs method, has been proposed in [41]. The basic idea is to fit a common meta-parameterization to the PDFs from different groups at some common scale $Q_0$, and then use the Monte Carlo combination of the different input sets to define the 68 % confidence-level intervals of these fit parameters. A Meta-PDF set combining MSTW08 [27], CT10 [18] and NNPDF2.3 [42] at NNLO was produced in [41] based on $N_\mathrm{eig}=50$ asymmetric eigenvectors. In addition, using the dataset diagonalization method proposed in [43], it is possible to further reduce the number of eigenvectors in the Meta-PDF sets for specific physical applications, such as for Higgs production processes.

The main limitation of the Meta-PDF method is the possible dependence on the choice of input meta-parametrization. Indeed, the statement that the common parameterization that is used to refit all PDF sets is flexible enough depends on which input sets enter in the combination, thus it needs to be checked and adjusted every time the procedure is repeated. In addition, at least for NNPDF, the Meta-PDF parameterization is bound to be insufficient, particularly in extrapolation regions like large-*x*, which are crucial for New Physics searches.

Recently, an alternative Hessian reduction approach, the MC2H method, has been developed [44]. This method adopts the MC replicas themselves as expansion basis, thus avoiding the need to choose a specific functional form. It uses Singular Value Decomposition methods with Principal Component Analysis to construct a representation of the PDF covariance matrix as a linear combination of MC replicas. The main advantage of the MC2H method is that the construction is exact, meaning that the accuracy of the new Hessian representation is only limited by machine precision. In practice, eigenvectors which carry little information are discarded, but even so with $N_\mathrm{eig}=100$ eigenvectors central values and covariances of the prior combination can be reproduced with $\mathcal {O}\left( 0.1\,\%\right)$ accuracy or better.

However, a central limitation of any Hessian reduction method is the impossibility of reproducing non-Gaussian features present in the input combination. It should be noted that even in the case where all the input sets in the combination are approximately Gaussian, their combination in general will be non-Gaussian. This is particularly relevant in extrapolation regions where PDF uncertainties are large and the underlying probability distributions for the PDFs are far from Gaussian. Failing to reproduce non-Gaussianities implies that the assumption of equal prior likelihood of the individual sets that enter the combination is artificially modified: for instance, if two sets peak at some value and another one at some other value (so we have a double hump structure), a Gaussian reduction effectively will be adding more weight to the second set as compared to the first two. To overcome this limitation is the main motivation for this work, where we propose an alternative reduction strategy based on the compression of the original Monte Carlo combined set into a smaller subset of replicas, which, however, reproduces the main statistical features of the input distribution.

The starting point of our method is, as in the case of the Meta-PDF and MC2H methods, the Monte Carlo combination of individual PDF sets, and then a compression algorithm follows in order to select a reduced number of replicas while reproducing the basic statistical properties of the original probability distribution, such as means, variances, correlations, and higher moments. This compression is based on the genetic algorithms (GA) exploration of the space of minima of suitably defined error functions, a similar strategy as that used for the neural network training in the NNPDF fits [45, 46]. The resulting compressed Monte Carlo PDFs, or CMC-PDFs for short, are then validated for a wide variety of LHC observables, both at the level of inclusive cross sections, differential distributions, and correlations, finding that using around $N_\mathrm{rep}=100$ replicas are enough to reproduce the original results for all the processes we have considered.

Another important application of the compression algorithm is to native Monte Carlo PDF sets. For instance, in the NNPDF framework, a large number of replicas, around $N_\mathrm{rep}=1000$, are required to reproduce fine details of the underlying probability distribution such as small correlations. Therefore, we can apply the same compression algorithm also to native MC PDF sets, and end up with a much smaller number of replicas conveying the same information as the original probability distribution. Therefore, in this work we will also present results of this compression of the NNPDF3.0 NLO $N_\mathrm{rep}=1000$ set. Note that despite the availability of the compressed sets, PDF sets with $N_\mathrm{rep}=1000$ replicas are still needed for other applications, for instance for Bayesian reweighting [47, 48].

The outline of this paper is as follows. First of all in Sect. 2 we review the Monte Carlo method for the combination of individual PDF sets, and we present results for the combination of the NNPDF3.0, MMHT14, and CT14 NNLO, both at the level of PDFs and for selected benchmark LHC cross sections. Then in Sect. 3 we describe the compression algorithm used to reduce the number of replicas of a MC PDF set. Following this, in Sect. 4 we present our main results for the CMC-PDFs, and validate our approach for the PDF central values, variances and correlations, together with selected parton luminosities. We also validate the compression of native MC sets, in particular using NNPDF3.0 NLO with $N_\mathrm{rep}=1000$ replicas. Then in Sect. 5 we perform the validation of the CMC-PDFs at the level of LHC cross sections and differential distributions. Finally, in Sect. 6 we summarize and discuss the delivery of our results, both for the CMC-PDFs to be made available in LHAPDF6 [49] and for the compression code, which is also made publicly available [50]. Appendix contains a concise user manual for the compression code, which allows construction of CMC-PDFs starting from an arbitrary input combination of PDF sets.

The detailed comparison of the CMC-PDFs with those of the Meta-PDF and MC2H methods will be presented in the upcoming PDF4LHC report with the recommendations about PDF usage at Run II.

## Combining PDF sets using the Monte Carlo method

In this section we review the Monte Carlo method for combination of different PDF sets, and we provide results for the combination of the recent NNPDF3.0, CT14, and MMHT14 NNLO PDF sets. We then compare this combined PDF set with the predictions from the three individual sets for a number of benchmark LHC inclusive cross sections and their correlations.

### Combination strategy

Our starting point is the same as that originally suggested by Forte in Ref. [1]. First of all we decide which sets enter the combination, then transform the Hessian sets into a Monte Carlo representation using the Watt and Thorne method [35] and finally combine the desired number of replicas from each set to construct the joint probability distribution of the combination. This strategy was already used in [2, 35, 36] to compare the predictions of the Monte Carlo combination of PDF sets with those of the original PDF4LHC envelope recommendation [24, 25].

Let us recall that a Monte Carlo representation for a Hessian set can be constructed [35] by generating a multi-Gaussian distribution in the space of fit parameters, with mean value corresponding to the best-fit result, and with width determined by the Hessian matrix. This is most efficiently done in the basis where the Hessian matrix is diagonal, and in this case Monte Carlo replicas can be generated using

1 $$\begin{aligned} F^k \!=\! F(q_0) \!+\! {{1}\over {2}} \sum _{j=1}^{N_\mathrm{eig}} \left[ F(q_j^+)\! -\! F(q_j^-) \right] \,R_j^k , \quad k=1,\ldots ,N_\mathrm{rep}, \end{aligned}$$

where $q_0$ and $q_j^\pm$ are, respectively, the best-fit and the asymmetric *j*th eigenvector PDF member, and $R_j^k$ are univariate Gaussian random numbers. For most practical applications, $N_\mathrm{rep}=100$ are enough to provide an accurate representation of the original Hessian set [35]. In this work we use the LHAPDF6 [49] implementation^1^ of Eq. (1). In particular, we use the LHAPDF6 program examples/hessian2replicas.cc to convert an entire Hessian set into its corresponding MC representation. In Eq. (1) the quantity *F* represents the value of a particular PDF at (*x*, *Q*) and flavors corresponding to the original LHAPDF6 grids.

Once Hessian PDF sets have been converted into their Monte Carlo representations, one needs to decide how many replicas $N_\mathrm{rep}^{(i)}$ of each PDF set *i* will be included in the combination. The combined probability distribution is simply $P = \sum _{i=1}^n w_i\,P_i$, where $P_i$ ($i=1,\ldots ,n$) are the probability distributions for each of the *n* individual PDF sets and the weights $w_i=N_\mathrm{rep}^{(i)}/\widetilde{N}_\mathrm{rep}$ ($i=1,\ldots ,n$), where $\sum _{i=1}^n w_i = 1$ and $\widetilde{N}_\mathrm{rep}=\sum _{i=1}^n N_\mathrm{rep}^{(i)}$ is the total number of replicas. The simplest case, corresponding to an equal degree of belief in the predictions from each of the PDF sets in the combination, is to use the same number of replicas, say $N_\mathrm{rep}^{(i)}=300$, from each set. This approach is justified in the case of fits based on a similar global dataset and comparable theory inputs, as will be the case in this work. Choosing the correct value of $N_\mathrm{rep}^{(i)}$ for sets based on a reduced dataset, or with very different theory inputs, is a more complex problem which is not discussed here. Note that taking the average over a large number of Monte Carlo replicas generated using Eq. (1) will recover the best-fit PDF member $F(q_0)$ only up to statistical fluctuations.

Using this Monte Carlo combination method, we have produced a combined set with $\widetilde{N}_\mathrm{rep}=900$ replicas from adding together $N_\mathrm{rep}^{(i)}=300$ replicas of the NNPDF3.0, CT14 and MMHT14 NNLO sets. Study of the properties of the prior with respect $\widetilde{N}_\mathrm{rep}$ shows that at least 900 replicas are required to eliminate the statistical fluctuations from Eq. (1) down to an acceptable level. For the three groups we use a common value of $\alpha _s(M_Z)=0.118$. One requirement for the validation of this procedure is that the combination of the same number of instances of *n* different probability distributions should have mean $\mu \approx {{1}\over {n}}\sum _{i=1}^n \mu _i$ and variance $\sigma ^2 \approx \sum _{i=1}^n \left( \mu _i^2 + \sigma ^2_i \right) /n - \mu ^2.$ The equality only holds when the three input distributions are Gaussian, which in the case of NNPDF is approximately true in the experimental data region.

**Fig. 1:**
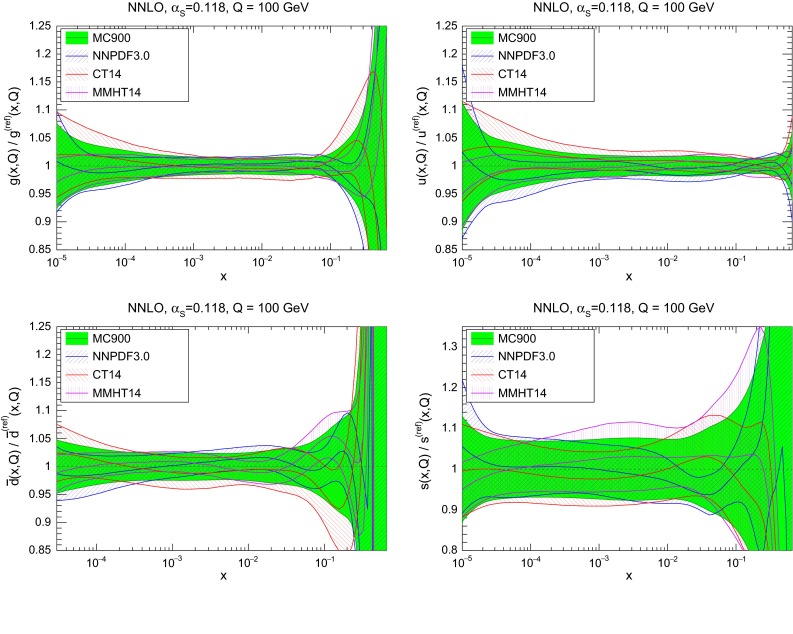
Comparison of the individual NNPDF3.0, CT14 and MMHT14 NNLO sets with the corresponding Monte Carlo combination MC900. The comparison is performed at a typical LHC scale of $Q=100$ GeV, and the PDFs are normalized to the central value of the combined set MC900

In this MC combination strategy, which is a common ingredient of the CMC-PDF, Meta-PDF, and MC2H methods, the theoretical inputs from each PDF group, like the method of solution of the DGLAP evolution equations, or the values of the heavy-quark masses, are not modified. Given that the current MC combination is based on PDF sets with different choices of the heavy-quark masses $m_c$ and $m_b$, and different heavy-quark schemes, for applications which depend sizably on the values of the heavy-quark masses and/or of the PDFs close to the heavy-quark thresholds, one should use the individual PDF sets rather than their combination. This might, however, change in future combinations if these are based on PDF sets with common settings for the treatment of heavy quarks.

While the starting point is common, the differences between the three reduction methods arises in the strategies adopted to decrease the number of error PDF sets in the combination, which is achieved by compressing the MC representation (CMC-PDFs) or by constructing a Hessian representation, based either on a meta-parametrization (Meta-PDFs) or in a linear expansion over the MC replicas themselves (MC2H). In the Meta-PDF approach [41], common theory settings are used to evolve upwards the meta-parameterization starting from $Q_0=8$ GeV using HOPPET [51], while CMC-PDF and MC2H maintain the original theory settings of each individual PDF set. It has been concluded, following a careful benchmarking between the two groups, that both options provide an adequate enough representation of the MC prior for $Q > m_b$, and in any case the current combined PDFs should not be used for $Q \lesssim m_b$.

In Fig. 1 we show the comparison of the individual PDF sets, NNPDF3.0, CT14, and MMHT14, with their Monte Carlo combination with $\widetilde{N}_\mathrm{rep}=900$. In the following, we will denote by MC900 this prior combination. The comparison is performed at a typical LHC scale of $Q=100$ GeV, and the PDFs are normalized to the central value of the combined set. As can be seen there is reasonable agreement between the three individual sets, and the resulting combined set is a good measure of their common overlap. Note that at large-*x* differences between the three sets are rather marked, and we expect the resulting combined probability distribution to be rather non-Gaussian.

**Fig. 2:**
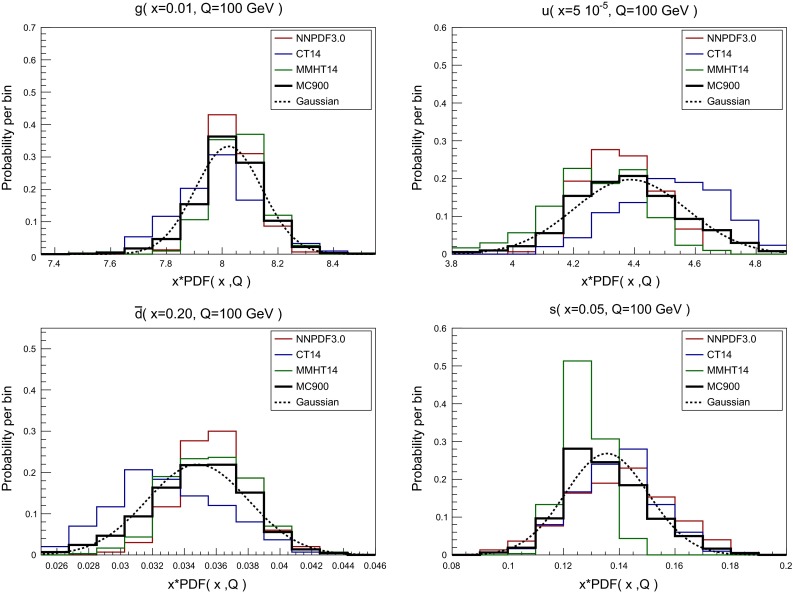
*Histograms* representing the probability distribution of Monte Carlo replicas for both the individual PDF sets and for the combined set, for different flavors and values of (*x*, *Q*). From *top* to *bottom* and from *left* to *right* we show the gluon at $x=0.01$, the up quark at $x=5\cdot 10^{-5}$, the down antiquark for $x=0.5$, and the strange PDF for $x=0.05$. All PDFs have been evaluated at $Q=100$ GeV. A Gaussian distribution computed with from the mean and variance of the MC900 prior is also shown

In Fig. 2 we show the histograms representing the distribution of Monte Carlo replicas in the individual PDF sets and in the combined set, for different flavors and values of (*x*, *Q*). From top to bottom and from left to right we show the gluon at $x=0.01$ (relevant for Higgs production in gluon fusion), the up quark at $x=5\cdot 10^{-5}$ (at the lower edge of the region covered by HERA data), the down antiquark for $x=0.2$ (relevant for high-mass searches) and the strange PDF for $x=0.05$ (accessible at the LHC through *W*+charm production). All PDFs have been evaluated at $Q=100$ GeV.

The histograms for the MC900 prior allow us to determine in each case how close the combined distribution is to a normal distribution, by comparison with a Gaussian computed using the same mean and variance of the MC900 set. From this comparison in Fig. 2, we see that while in some cases the underlying distribution of the MC900 PDFs is reasonably Gaussian, like for $g(x=0.01)$ and $u(x=5\cdot 10^{-5})$, in others, for $\bar{d}(x=0.2)$ and $s(x=0.05)$, the Gaussian approximation is not satisfactory. Deviations from a Gaussian distribution are in general more important for PDFs in extrapolation regions with limited experimental information.

**Fig. 3:**
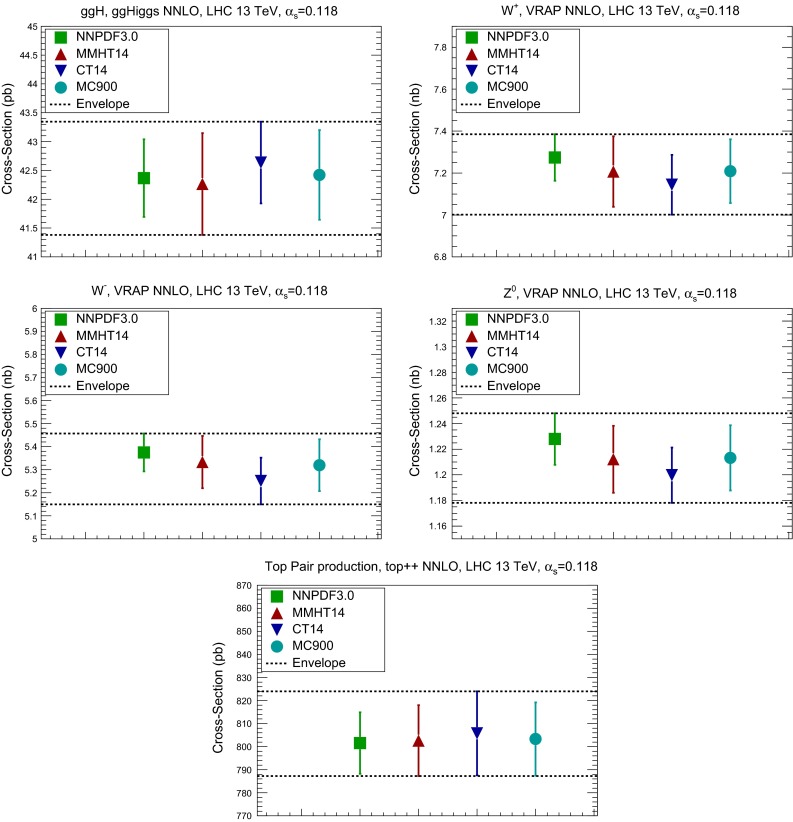
Comparison of the predictions from the NNPDF3.0, MMHT14 and CT14 NNLO sets, with those of their Monte Carlo combination MC900, for a number of inclusive benchmark LHC cross sections. For illustration, we also indicate the envelope of the predictions of the three different PDF sets, which would determine the total PDF uncertainty in the current PDF4LHC recommendation. From *top* to *bottom* and from *left* to *right*: Higgs production in gluon fusion, $W^+$, $W^-$, and *Z* production, and *top* quark pair production. All processes have been computed at the LHC with a center-of-mass energy of 13 TeV

Concerning the treatment of the PDF+$\alpha _s$ uncertainties, the updated PDF4LHC recommendation [33] proposes a simplified prescription based on the addition in quadrature of the separated $\delta \sigma ^\mathrm{PDF}$ and $\delta \sigma ^{\alpha _s}$ uncertainties, based on the realization that this always gives approximately the same answer as more sophisticated methods, and in some procedures exactly the same answer. In the case of the Monte Carlo combination, this prescription can be implemented by simply constructing the central values of the MC900 prior with, say, $\alpha _s(M_Z)=0.1165$ and $\alpha _s(M_Z)=0.1195$ as the mean of the central values of the NNPDF3.0, MMHT14, and CT14 sets, each with the corresponding value of $\alpha _s(M_Z)$. Half of the spread of the predictions computed with the central values of the CMC-PDFs with $\alpha _s(M_Z)=0.1165$ and $\alpha _s(M_Z)=0.1195$ defines then the one-sigma $\delta \sigma ^{\alpha _s}$ uncertainty. This assumes $\alpha _s(M_Z)=0.118\pm 0.0015$ as an external input, but a different value of $\delta \alpha _s$ can be implemented by a simple rescaling. Note also that for the MC900 sets with $\alpha _s(M_Z)\ne 0.118$, only the central values are required.

**Fig. 4:**
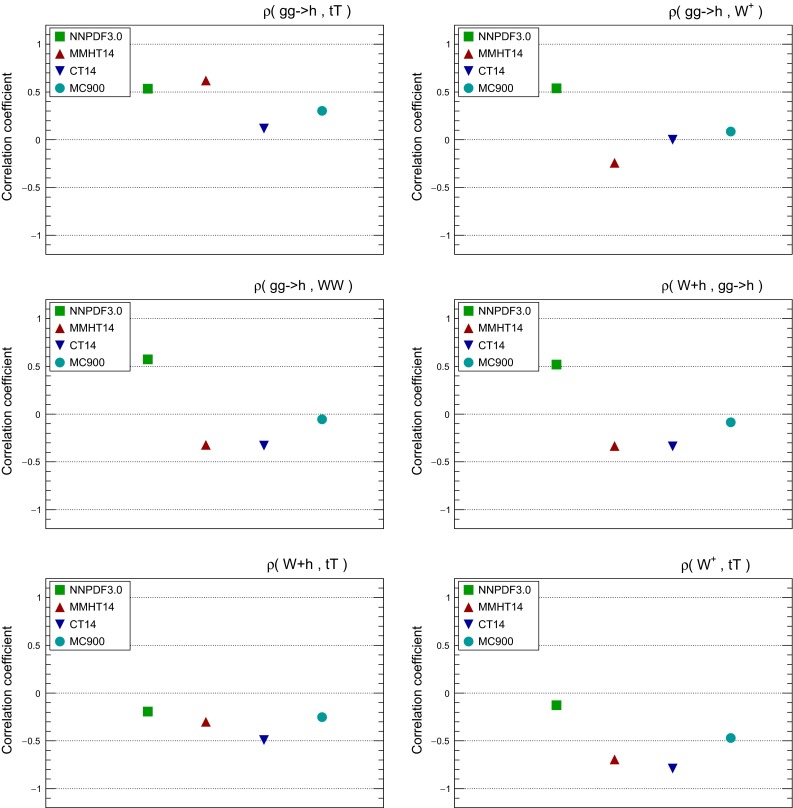
Comparison of the correlation coefficients between a number of representative NLO and NNLO LHC inclusive cross sections computed from the three individual sets, NNPDF3.0, CT14, and MMHT14 (using the MC representation for the Hessian sets), and with their MC combination MC900

### PDF dependence of benchmark LHC cross sections

As stated in the introduction, the goal of this work is to compress the MC900 prior by roughly an order of magnitude, from the starting $\widetilde{N}_\mathrm{rep}=900$ to at least $N_\mathrm{rep}\simeq 100$, and to validate the results of this compression for a number of LHC observables. In Sect. 4 we will show the results of applying the compression strategy of Sect. 3 to the combined MC set. But first let us explore how the predictions from MC900 prior compare with the individual PDF sets for a variety of LHC cross sections. We also compare the correlations between physical observables for the individual PDF sets to their combination.

In the following we consider a number of NNLO inclusive cross sections: Higgs production in gluon fusion, computed using ggHiggs [52], top-quark pair production, using top++ [53], and inclusive *W* and *Z* production, using VRAP [54]. In all cases we use the default settings in each of these codes, since our goal is to study similarities and differences between the predictions of each of the PDF sets, for fixed theory settings.

The results for these inclusive cross sections are shown in Fig. 3. We also show with dashed lines the envelope of the one-sigma range obtained from the three individual sets, which would correspond to the total PDF uncertainty for this process if obtained following the present PDF4LHC recommendation. We see that in general the two methods, the MC combination and the envelope, give similar results, the former leading to a smaller estimate of the total PDF uncertainty since the envelope assigns more weight to outliers than what would be required on a statistical basis.

It is also useful to compare the correlations between LHC cross sections computed with the individual PDF sets and with the MC900 combined set. A representative set of these correlations is shown in Fig. 4, computed using the same settings as above. In addition to the processes shown in Fig. 3, here we also show correlations for the *WW* and *Wh* production NLO total cross sections computed with MFCM. For MMHT14 and CT14, correlations are computed from their Monte Carlo representation.

From the comparison of the correlation coefficients shown in Fig. 4 we note that the correlation coefficients between LHC cross sections for the three global sets, NNPDF3.0, CT14, and MMHT14, can differ substantially more than for central values and variances. This effect was also noticed in the Higgs Cross-Section Working Group study of PDF-induced correlations between Higgs production channels [55]. By construction, the correlation coefficient for the combined MC prior produces the correct weighted average of the correlations from the individual sets.

## The compression algorithm

In the previous section we have described and validated the combination of different PDF sets, based on the Monte Carlo method. We have shown that the probability distribution of such a combined PDF set can be represented by $\widetilde{N}_\mathrm{rep}$ Monte Carlo replicas. Now in this section we introduce a compression algorithm that aims to determine, for a fixed (smaller) number of MC replicas $N_\mathrm{rep} < \widetilde{N}_\mathrm{rep}$, the optimal subset of the original representation that most faithfully reproduces the statistical properties of the combined PDF prior distribution.

First of all, we begin with a presentation of the mathematical problem, followed by a description of the technical aspects of the compression strategy, where we describe the choice of error function and related parameters that have been chosen in this work. Then we apply the compression method to the combined Monte Carlo PDFs, producing what will be dubbed as CMC-PDFs in the rest of this paper. We also show how the compression strategy can be applied to native Monte Carlo PDF sets, using the NNPDF3.0 NLO set with $\widetilde{N}_\mathrm{rep}=1000$ as an illustration. The validation of the compression at the level of parton distributions and physical observables is then performed in Sects. 4 and 5.

### Compression: mathematical framework

Let us begin by presenting an overview of the mathematical framework for the problem that we aim to address, namely the compression of a given probability distribution function. The starting point is to consider a representation of a probability distribution ${\vec {p}}=(p_1,\ldots ,p_n)$, using a finite number *n* of instances. In the case at hand, the number of instances is given by the number of Monte Carlo replicas $\widetilde{N}_\mathrm{rep}$. Any smaller number set of replicas, $N_\mathrm{rep} < \widetilde{N}_\mathrm{rep}$, produces a corresponding probability distribution ${\vec {q}}$, which entails a loss of information with respect to the original distribution ${\vec {p}}$. The problem of optimal compression can be mathematically stated as follows. We would like to find the specific subset of the original set of replicas such that the statistical distance between the original and the compressed probability distributions is minimal. In other words, we look for a subset of replicas that delivers a probability distribution as indistinguishable from the prior set as possible.

A number of different figures of merit to quantify the distinguishability of probability distribution were proposed many decades ago. Some of the first efforts are accounted in the book of Hardy et al. [56], where ideas about strong ordering (majorization) were introduced. Later on, the problem of distinguishability was quantified using the concept of statistical distance among probability distributions. In particular, the Kolgomorov distance

2 $$\begin{aligned} K({\vec {p}}, {\vec {q}}) = \sum _i |p_i-q_i|, \quad i=1,\ldots ,n , \end{aligned}$$

where the index *i* runs over the number of instances of $\vec {p}$, is a simple and powerful example of a figure of merit that quantifies how different a probability distribution is from another one.

With the advent of Information Theory, Shannon introduced the concept of *surprise* of a probability distribution as its *distance* to the even prior. This can be characterized using Shannon entropy $S({\vec {p}})=-\sum _i p_i \log p_i$. It is, then, natural to quantify distinguishability between two probability distributions ${\vec {p}}$ and ${\vec {q}}$ using entropy concepts [57]. This leads to the construction of the Kullback–Leibler divergence

3 $$\begin{aligned} D({\vec {p}} || {\vec {q}})= \sum _{i=1,\ldots ,n} q_i \log {{q_i}\over {p_i}}, \end{aligned}$$

which differs from the Kolmogorov distance in the sense that it weights more the largest probabilities. Later refinements produced the ideas of symmetric statistical distances, like the symmetrized Kullback and the Chernhoff distances, used in Quantum Information nowadays. As a consequence of these trend of ideas, it is clear there are very many well-studied options to define a distance in probability space. Since their variations are not large, any of them should be suitable for the problem of Monte Carlo PDF compression, and we present our specific choice in Sect. 3.2.

Let us now be more precise on the way we shall proceed. If we define $\{\vec {p}\}$ as the original representation of the probability distribution (with $\widetilde{N}_\mathrm{rep}$ replicas) and $\{\vec {q}\}$ its compressed version (with $N_\mathrm{rep}$ replicas), then given the concept of a distance *d* between two probability distributions there is an optimal choice of the subset with $N_\mathrm{rep}$ replicas defined as

4 $$\begin{aligned} \{\vec {q}\}_\mathrm{opt} \equiv \mathrm{Min}_{\{\vec {q}\}} \left[ d \left( \{\vec {q}\},\{\vec {p}\} \right) \right] . \end{aligned}$$

Therefore, the mathematical problem at stake is reduced to finding the optimal subset $\{\vec {q}\}_\mathrm{opt}$, by a suitable exploration of the space of minima of the distance $d \left( \{\vec {q}\},\{\vec {p}\} \right)$. In this work, this exploration is performed using genetic algorithms, though many other choices would also be suitable. Fortunately, many choices of subset are equally good minimizations. From the practical point of view, the specific choice of the minimization strategy is not critical. It is clear that the relevant point is the definition of a distance between the original and compressed replica sets. In this paper we shall take the following approach.

Many valid definitions of statistical distance differ in the way different moments are weighted. Since we are interested in reproducing real physics, which is dominated by low moments, we shall explicitly include in our figure of merit all the distances between means and standard deviations, but also kurtosis, skewness and correlations, as well as higher moments. As a consequence, all of them will be minimized, favoring the role of smaller moments.

### A compression algorithm for Monte Carlo PDF sets

As we have discussed above, the most important ingredient for the compression strategy is the choice of a suitable distance between the prior and the compressed distributions, Eq. (4), or in other words, the definition of the error function (ERF in the following) for the minimization problem. We have explored different possibilities, and the precise definition of the ERF that will be used in this work can be written generically as follows:

5 $$\begin{aligned} \mathrm{ERF}=\sum _{k} {{1}\over {N_{k}}} \sum _{i}\left( {{C^{(k)}_{i}-O^{(k)}_{i}}\over {O^{(k)}_{i}}}\right) ^{2}, \end{aligned}$$

where *k* runs over the number of statistical estimators used to quantify the distance between the original and compressed distributions, $N_{k}$ is a normalization factor, $O^{(k)}_{i}$ is the value of the estimator *k* (for example, the mean or the variance) computed at the generic point *i* (which could be a given value of (*x*, *Q*) in the PDFs, for instance), and $C^{(k)}_{i}$ is the corresponding value of the same estimator in the compressed set. The choice of a normalized ERF is important for the accuracy of the minimization because some statistical estimators, in particular higher moments, can span various orders of magnitude in different regions of *x* and $Q^2$.

**Fig. 5:**
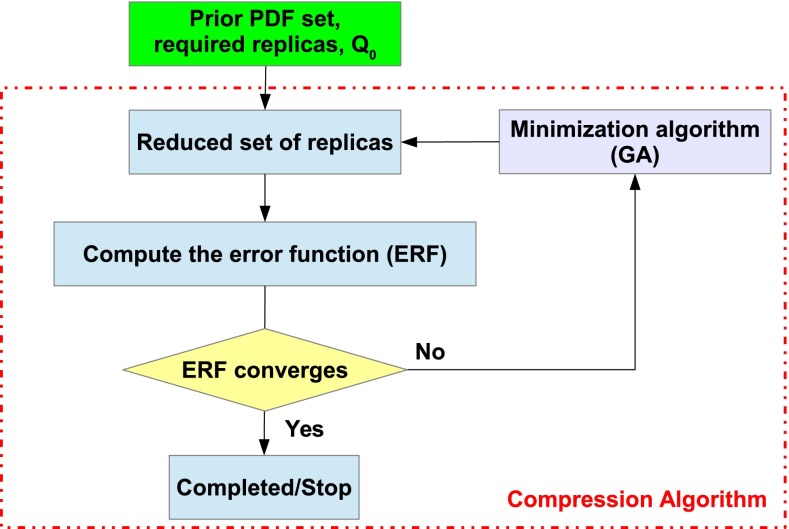
Schematic representation of the compression strategy used in this work: a prior PDF set and the number of compressed replicas is the input of a GA algorithm which selects the best subset of replicas which minimizes the ERF between the prior and the compressed set

An schematic diagram for our compression strategy is shown in Fig. 5. The prior set of Monte Carlo PDF replicas, the desired number of compressed replicas, $N_\mathrm{rep}$, and the value of the factorization scale *Q* at which the PDFs are evaluated, $Q_0$, are the required parameters for the compression algorithm. Note that it is enough to sample the PDFs in a range of values of Bjorken-*x* at a fixed value of $Q_0$, since the DGLAP equation uniquely determines the evolution for higher scales $Q \ge Q_0$. The minimization of the error function is performed using genetic algorithms (GAs), similarly as in the neural network training of the NNPDF fits. GAs work as usual by finding candidates for subsets of $N_\mathrm{rep}$ leading to smaller values of the error function Eq. (5) until some suitable convergence criterion is satisfied. The output of this algorithm is thus the list of the $N_\mathrm{rep}$ replicas from the prior set of $\widetilde{N}_\mathrm{rep}$ that minimize the error function. These replicas define the CMC-PDFs for each specific value of $N_\mathrm{rep}$. The final step of the process is a series of validation tests where the CMC-PDFs are compared to the prior set in terms of parton distributions at different scales, luminosities, and LHC cross sections, in a fully automated way.

It is important to emphasize that the compression algorithm only selects replicas from a prior set, and no attempt is made to use common theoretical settings, i.e., the method for the solution of the DGLAP evolution equations, or the values of the heavy-quark masses, which are those of the corresponding original PDF sets. This important fact automatically ensures that the compressed set conserves all basic physical requirements of the original combined set such as the positivity of physical cross sections, sum rules, and the original correlations between PDFs. To avoid problems related to the different treatment of the heavy-quark thresholds between the different groups, we choose in this work to compress the combined MC PDF set at a common scale of $Q_0=2$ GeV, while we use $Q_0=1$ GeV when compressing the native NNPDF3.0 NLO set.

The compression strategy seems conceptually simple: reducing the size of a Monte Carlo PDF set requiring no substantial loss of information. In order to achieve its goal, the compression algorithm must preserve as much as possible the underlying statistical properties of the prior PDF set. However, this conceptual simplicity is followed by a series of non-trivial issues that have to be addressed in the practical implementation. Some of these issues are the sampling of the PDFs in Bjorken-*x*, the exact definition of the error function, Eq. (5), the treatment of PDF correlations and the settings of the GA minimization. We now discuss these various issues in turn.

#### Definition of the error function for the compression

In this work we include in the ERF, Eq. (5), the distances between the prior and the compressed sets of PDFs for the following estimators:

- The first four moments of the distribution, which are sampled in a grid of *x* points for $n_{f}$ flavors in terms of central value, standard deviation, skewness, and kurtosis, at a fixed value of $Q=Q_0$. It is important to notice that these estimators are necessary in order to obtain a realistic and optimized compressed MC set, but are not sufficient to avoid eventual bias of continuity and loss of structure.
- The output of the Kolmogorov–Smirnov test. This is the simplest distance between empirical probability distributions. This distance complements the terms in the ERF which contain the first four moments, by ensuring that also higher moments are automatically adjusted. However, if this estimator is used alone possible ambiguities arise when defining the regions where the distance is computed, leading to large errors when working with few replicas.
- The correlation between multiple PDF flavors at different *x* points. This information is important for ensuring that PDF-induced correlations in physical cross sections are successfully maintained.

The final figure of merit used in the compression fit is then the sum over all these six estimators opportunely weighted by the corresponding normalization factors $N_{k}$ in Eq. (5). This normalization is required due to the fact that the absolute value of the various estimators can vary among them by several orders of magnitude.

#### Central values, variances, and higher moments

Let’s denote by $g_{i}^{(k)}(x_{j},Q_0)$ and $f_{i}^{(r)}(x_{j},Q_0)$, respectively, the prior and the compressed sets of replicas for a flavor *i* at the position *j* of the *x*-grid containing $N_{x}$ points. $N_{\mathrm{rep}}$ is the number of required compressed replicas. We then define the contribution to the ERF from the distances between central values of the prior and compressed distributions as follows:

6 $$\begin{aligned} \mathrm{ERF}_\mathrm{CV}\!=\!{{1}\over {N_{\mathrm{CV}}}}\sum _{i=-n_{f}}^{n_{f}}\sum _{j=1}^{N_{x}}\left( {{f_{i}^{\mathrm{CV}}(x_{j},Q_0)-g_{i}^{\mathrm{CV}}(x_{j},Q_0)}\over {g_{i}^{\mathrm{CV}}(x_{j},Q_0)}}\right) ^{2}, \end{aligned}$$

where $N_\mathrm{CV}$ is the normalization factor for this estimator. We only include in the sum those points for which the denominator satisfies $g_{i}^\mathrm{CV}(x_{j},Q_0)\ne 0$. As usual, central values are computed as the average over the MC replicas, for the compressed set

7 $$\begin{aligned} f_{i}^{\mathrm{CV}}(x_{j},Q_0)={{1}\over {N_{\mathrm{rep}}}} \sum _{r=1}^{N_{\mathrm{rep}}}f_{i}^{(r)}(x_{j},Q_0), \end{aligned}$$

while for the prior set we have

8 $$\begin{aligned} g_{i}^{\mathrm{CV}}(x_{j},Q_0)={{1}\over {\widetilde{N}_{\mathrm{rep}}}}\sum _{k=1}^{\widetilde{N}_{\mathrm{rep}}}g_{i}^{(k)}(x_{j},Q_0) . \end{aligned}$$

Let us also define $r^{t}_{i}(x_{j},Q_0)$ as a random set of replicas extracted from the prior set, where *t* identifies an ensemble of random extractions. The number of random extraction of random sets is denoted by $N_{\mathrm{rand}}$.

Now, the normalization factors are extracted for all estimators as the lower 68 % confidence-level value obtained after $N_\mathrm{rand}$ realizations of random sets. In particular for this estimator we have

9 $$\begin{aligned} N_{\mathrm{CV}}&={{1}\over {N_{\mathrm{rand}}}}\sum _{d=1}^{N_{\mathrm{rand}}}\sum _{i=-n_{f}}^{n_{f}}\sum _{j=1}^{N_{x}}\nonumber \\&\quad \times \left. \left( {{r_{i}^{d,\mathrm{CV}}(x_{j},Q_0)-g_{i}^{\mathrm{CV}}(x_{j},Q_0)}\over {g_{i}^{\mathrm{CV}}(x_{j},Q_0)}}\right) ^{2}\right| _{{\mathrm{68\,\%\,lower\,band}}}. \end{aligned}$$

For the contribution to the ERF from the distance between standard deviation, skewness, and kurtosis, we can build expressions analogous to that of Eq. (6) by replacing the central value estimator with the suitable expression for the other statistical estimators, which in a Monte Carlo representation can be computed as

10 $$\begin{aligned}&f_{i}^{\mathrm{STD}}(x_{j},Q_0)\nonumber \\&\quad =\sqrt{{{1}\over {N_{\mathrm{rep}}-1}}\sum _{r=1}^{N_{\mathrm{rep}}}\left( f_{i}^{(r)}(x_{j},Q_0)-f_{i}^{\mathrm{CV}}(x_{j},Q_0)\right) ^{2}}, \end{aligned}$$

11 $$\begin{aligned}&f_{i}^{\mathrm{SKE}}(x_{j},Q_0)={{1}\over {N_{\mathrm{rep}}}}\sum _{r=1}^{N_{\mathrm{rep}}}\nonumber \\&\quad \times \left( f_{i}^{(r)}(x_{j},Q_0)-f_{i}^{\mathrm{CV}}(x_{j},Q_0)\right) ^{3}\Big /\left( f_{i}^{\mathrm{STD}}(x_{j},Q_0)\right) ^{3},\end{aligned}$$

12 $$\begin{aligned}&f_{i}^{\mathrm{KUR}}(x_{j},Q_0)={{1}\over {N_{\mathrm{rep}}}}\sum _{r=1}^{N_{\mathrm{rep}}}\nonumber \\&\quad \times \left( f_{i}^{(r)}(x_{j},Q_0)-f_{i}^{\mathrm{CV}}(x_{j},Q_0)\right) ^{4}\Big /\left( f_{i}^{\mathrm{STD}}(x_{j},Q_0)\right) ^{4},\nonumber \\ \end{aligned}$$

for the compressed set, with analogous expressions for the original prior set.

The normalization factors for these estimators are extracted using the same strategy presented in Eq. (9), by averaging over random extractions of $N_\mathrm{rep}$ replicas, exchanging CV by STD, SKE, and KUR, respectively.

#### The Kolmogorov–Smirnov distance

As we have mentioned above, the minimization of the Kolmogorov–Smirnov distance ensures that both lower and higher moments of the prior distribution are successfully reproduced. In our case, we define the contribution to the total ERF from the Kolmogorov–Smirnov (KS) distance as follows:

13 $$\begin{aligned} \mathrm{ERF}_\mathrm{KS}\!=\!{{1}\over {N_\mathrm{KS}}}\sum _{i=-n_{f}}^{n_{f}}\sum _{j=1}^{N_{x}}\sum _{k=1}^{(r)}\left( {{F_{i}^{k}(x_{j},Q_0)-G_{i}^{k}(x_{j},Q_0)}\over {G_{i}^{k}(x_{j},Q_0)}}\right) ^{2}. \end{aligned}$$

where $F^k_i(x_j,Q_0)$ and $G^k_i(x_j,Q_0)$ are the outputs of the test for the compressed and the prior set of replicas, respectively. The output of the test consists in counting the number of replicas contained in the *k* regions where the test is performed. We count the number of replicas which fall in each region and then we normalize by the total number of replicas of the respective set. Here we have considered six regions defined as multiples of the standard deviation of the distribution for each flavor *i* and $x_{j}$-point. As an example for the compressed set, the regions are

14 $$\begin{aligned}&\big [-\infty ,-2f_{i}^{\mathrm{STD}}(x_{j},Q_0),-f_{i}^{\mathrm{STD}}(x_{j},Q_0),0,f_{i}^{\mathrm{STD}}(x_{j},Q_0),\nonumber \\&\quad 2f_{i}^{\mathrm{STD}}(x_{j},Q_0),+\infty \big ], \end{aligned}$$

where the values of the PDFs have been subtracted from the corresponding central value.

In this case, the normalization factor is determined from the output of the KS test for random sets of replicas extracted from the prior, denoted $R^k_i(x_j,Q_0)$ as follows:

15 $$\begin{aligned} N_\mathrm{KS}&={{1}\over {N_{\mathrm{rand}}}}\sum _{d=1}^{N_{\mathrm{rand}}}\sum _{i=-n_{f}}^{n_{f}}\sum _{j=1}^{N_{x}}\sum _{k=1}^{6}\nonumber \\&\quad \times \left( {{R_{i}^{k}(x_{j},Q_0)-G_{i}^{k}(x_{j},Q_0)}\over {G_{i}^{k}(x_{j},Q_0)}}\right) ^{2}, \end{aligned}$$

and we only include in the sum those points for which the denominator satisfies $G_{i}^{k}(x_{j},Q_0)\ne 0$.

#### PDF correlations

In addition to all the moments of the prior distribution, a sensible compression should also maintain the correlations between values of *x* and between flavors of the PDFs. In order to achieve this, correlations are taken into account in the ERF by means of the trace method. We define a correlation matrix *C* for any PDF set as follows:

16 $$\begin{aligned} C_{ij}={{N_\mathrm{rep}}\over {N_\mathrm{rep}-1}}\cdot {{\langle ij\rangle -\langle i\rangle \langle j\rangle }\over {\sigma _{i}\cdot \sigma _{j}}}, \end{aligned}$$

where we have defined

17 $$\begin{aligned} \begin{aligned}&\langle i\rangle ={{1}\over {N_{\mathrm{rep}}}}\sum _{r=1}^{N_{\mathrm{rep}}}f_{i}^{(r)}(x_{i},Q_0),\\&\langle ij\rangle ={{1}\over {N_{\mathrm{rep}}}}\sum _{r=1}^{N_{\mathrm{rep}}}f_{i}^{(r)}(x_{i},Q_0)f_{j}^{(r)}(x_{j},Q_0), \end{aligned} \end{aligned}$$

and $\sigma$ is the usual expression for the standard deviation

18 $$\begin{aligned} \sigma _{i}=\sqrt{{{1}\over {N_{\mathrm{rep}}-1}}\sum _{r=1}^{N_{\mathrm{rep}}}\left( f_{i}^{(r)}(x_{i},Q_0)-\langle i\rangle \right) ^{2}}. \end{aligned}$$

Now, for each flavor $n_{f}$ we define $N^\mathrm{corr}_{x}$ points distributed in *x* where the correlations are computed. The trace method consists in computing the correlation matrix *P* based on Eq. (16) for the prior set and then store its inverse $P^{-1}$. For $n_{f}$ flavors and $N^\mathrm{corr}_{x}$ points we obtain

19 $$\begin{aligned} g=\mathrm{Tr}(P\cdot P^{-1})= N^\mathrm{corr}_{x}\cdot (2 \cdot n_{f} + 1). \end{aligned}$$

After computing the correlation matrix for prior set, for each compressed set a matrix *C* is computed and the trace is determined by

20 $$\begin{aligned} f=\mathrm{Tr}(C\cdot P^{-1}). \end{aligned}$$

The compression algorithm then includes the correlation ERF by minimizing the quantity:

21 $$\begin{aligned} \mathrm{ERF}_\mathrm{Corr}={{1}\over {N_\mathrm{Corr}}}\left( {{f-g}\over {g}}\right) ^{2} \end{aligned}$$

where $N_\mathrm{Corr}$ is computed as usual from the random sets, in the same way as Eq. (9).

#### Choice of GA parameters in compressor v1.0.0

The general strategy that has been presented in this section has been implemented in compressor v1.0.0, the name of the public code [50] released together with this paper. A more detailed description of the code usage is provided in the appendix. The availability of this code ensures that it will be possible to easily redo the compression for any further combination of PDF sets that might be considered in the future.

**Table 1:** (a) Setting of the compression algorithm used in this work. (b) Mutation rates used in the genetic algorithm minimization

compressor v1.0.0	
(a) GA Parameters	
$N_\mathrm{gen}^{\max }$	15,000
$N_\mathrm{mut}$	5
$N_{x}$	70
$x_{\min }$	$10^{-5}$
$x_{\max }$	0.9
$n_{f}$	7
$Q_0$	User-defined
$N^\mathrm{corr}_{x}$	5
$N_\mathrm{rand}$	1000

(b)	
$N_\mathrm{rep}^\mathrm{mut}$	$P_\mathrm{mut}$ (%)
1	30
2	30
3	10
4	30

This said, there is a certain flexibility in the choice of settings for the compression, for example in the choice of parameters for the genetic algorithms, the sampling of the PDFs in *x* or the choice of common scale for the compression $Q_0$. The compression setup used in this paper is presented in Table 1 together with the optimal set of GA parameters and mutation probability rates, determined by trial and error.

As mentioned before, in the present work the compression of CMC-PDFs is performed at a scale of $Q_0=2$ GeV while in the next section we use $Q_0=1$ GeV for the native NNPDF3.0 NLO set. The ERF includes only the contribution of the $n_f=7$ light partons: *u*, $\bar{u}$, *d*, $\bar{d}$, *s*, $\bar{s}$, and *g*. Concerning the sampling of the PDFs in *x*, we have limited the range of *x* points to the region where data is available, i.e. $x\sim [10^{-5},0.9]$, by selecting 35 points logarithmically spaced between $[10^{-5}, 0.1]$ and 35 points linearly spaced from [0.1, 0.9]. Note that this is different from the Meta-PDF approach, where for each PDF a different range $\left[ x_{\min }, x_{\max }\right]$ is used for the fit with the meta-parametrization, restricted to the regions where experimental constraints are available for each flavor.

The correlation matrix is then computed for the $n_f$ input PDFs in $N^\mathrm{corr}_{x}=5$ points in *x*, generating a correlation matrix of 35 entries. Increasing the number of points for the calculation of the correlation matrix would be troublesome since numerical instabilities due to the presence of large correlations between neighboring points in *x* would be introduced.

The genetic algorithm minimization is performed for a fixed length of 15*k* generations. Note that as opposed to the neural network learning in the NNPDF fits, in the compression problem there is no risk of over-learning, since the absolute minimum of the error function always exists. On the other hand, we find that after a few thousand generations the ERF saturates and no further improvements are achieving by running the code longer, hence the maximum number of GA generations $N_\mathrm{gen}^{\max }=15k$ used in this work.

**Fig. 6:**
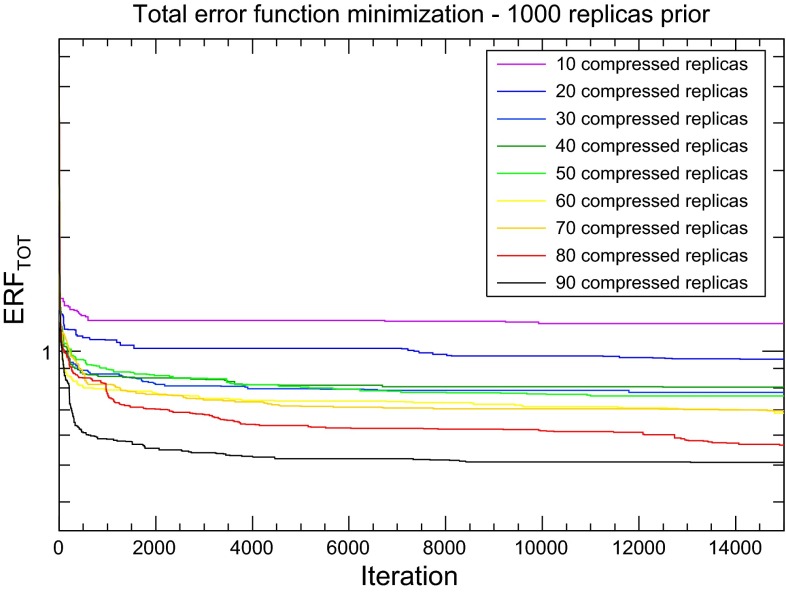
The value of the total error function, Eq. (5), for the compression of the 1000 replica set of NNPDF3.0 NLO, as a function of the number of GA generations, for different values of the number of replicas in the compressed set $N_\mathrm{rep}$. After 15*k* iterations, the error function saturates and no further improvement of the error function would be achieved for longer training

### Results of the compression for native MC PDF sets

In order to illustrate the performance of the compression algorithm, we consider here the compression of a native Monte Carlo set of PDFs at $Q_0=1$ GeV, based on the prior set with $\widetilde{N}_\mathrm{rep}=1000$ replicas of NNPDF3.0 NLO.

In Fig. 6 we show the dependence of the total ERF as a function of the number of iterations of the GA for $N_\mathrm{rep}=10,20,30,40,50,60,70,80$, and 90. We observe that the first 1*k* iterations are extremely important during the minimization, while after 15*k* iterations the total error function is essentially flat for any required number of compressed replicas. For each compression, the final value of the error function is different, with deeper minima being achieved as we increase the number of compressed replicas, as expected. The flatness of the ERF as a function of the number of iterations confirms that the current parameters provide a suitably efficient minimization strategy.

**Fig. 7:**
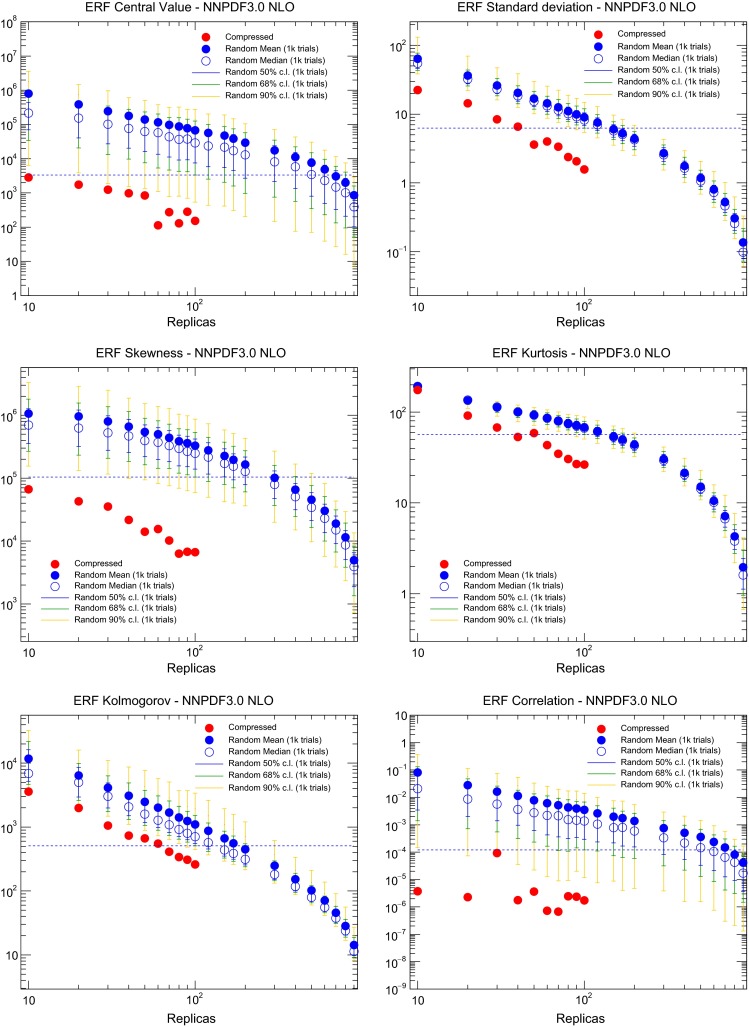
The various contributions to the ERF, Eq. (5), for the compression of the NNPDF3.0 NLO set with $\widetilde{N}_\mathrm{rep}=1000$ replicas. For each value of $N_\mathrm{rep}$, we show the value of each contribution to the ERF for the best-fit result of the compression algorithm (*red points*). We compare the results of the compression with the values of the ERF averaged over $N_\mathrm{rand}=1000$ random partitions of $N_\mathrm{rep}$ replicas (*blue points*), as well as the 50, 68, and 90 % confidence-level intervals computed over these random partitions. The dashed horizontal line is the 68 % lower band of the ERF for the average of the random partitions with $N_\mathrm{rep}=100$, and is inserted for illustration purposes only

In order to quantify the performance of the compression algorithm, and to compare it with that of a random selection of the reduced set of replicas, Fig. 7 shows the various contributions to the ERF, Eq. (5), for the compression of the NNPDF3.0 NLO set with $\widetilde{N}_\mathrm{rep}=1000$ replicas. For each value of $N_\mathrm{rep}$, we show the value of each contribution to the ERF for the best-fit result of the compression algorithm (red points). We compare the results of the compression with the values of the ERF averaged over $N_\mathrm{rand}=1000$ random partitions of $N_\mathrm{rep}$ replicas (blue points), as well as the 50, 68, and 90 % confidence-level intervals computed over these random partitions.

Various observations can be made from the inspection of Fig. 7. First of all, the various contributions to the ERF tend to zero when the number of compressed or random replicas tends to the size of the prior set, as expected for consistency. For the random partitions of $N_\mathrm{rep}$ replicas the mean value and the median values averaged over $N_\mathrm{rand}$ trials are not identical, emphasizing the importance of taking confidence levels. From Fig. 7 we also confirm that the compression algorithm is able to provide sets of PDFs with smaller ERF values for all estimators that outperform random selections with a much larger number of replicas. To emphasize this point, the dashed horizontal line in Fig. 7 corresponds to the lower limit of the 68 % confidence level of the ERF computed over $N_\mathrm{rand}=1000$ random partitions with $N_\mathrm{rep}=100$, and is inserted for illustration purposes only. It indicates that the NNPDF3.0 NLO PDF set with $\widetilde{N}_\mathrm{rep}=1000$ can now be compressed down to $N_\mathrm{rep}=50$ replicas in a way that reproduces better the original distribution that most of the random partitions of $N_\mathrm{rep}=100$ replicas.

The results of Fig. 7 confirm that the compression algorithm outperforms essentially any random selection of replicas for the construction of a reduced set, and provides an adequate representation of the prior probability distribution with a largely reduced number of replicas. Similar results are obtained when compressing the CMC-PDFs, as we will discuss in Sect. 4.2.

**Fig. 8:**
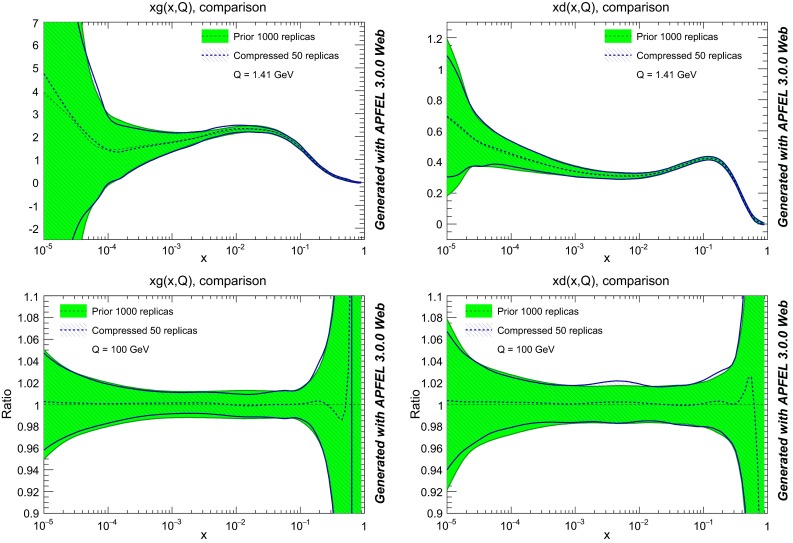
*Upper plots* comparison of the prior NNPDF3.0 NLO set with $\widetilde{N}_\mathrm{rep}=1000$ and the compressed set with $N_\mathrm{rep}=50$ replicas, for the gluon and the down quark at the scale $Q^2=2$ GeV$^2$. *Lower plots* the same comparison this time at a typical LHC scale of $Q=100$ GeV, normalized to the central value of the prior set

## The compressed Monte Carlo PDF sets

In this section we present the results for the CMC-PDFs, first discussing the compression of a native Monte Carlo PDF set, in this case NNPDF3.0 with $\widetilde{N}_\mathrm{rep}=1000$, and then the compression of the MC combination for NNPDF3.0, CT14, and MMHT14 with $\widetilde{N}_\mathrm{rep}=900$. In both cases, we compare the PDFs from the prior and compressed sets, for different values of the number of replicas $N_\mathrm{rep}$ of the latter. We also verify that correlations between PDFs are successfully reproduced by the compression. The phenomenological validation of the CMC-PDF sets at the level of LHC observables is addressed in Sect. 5.

### Compression of native MC PDF sets

**Fig. 9:**
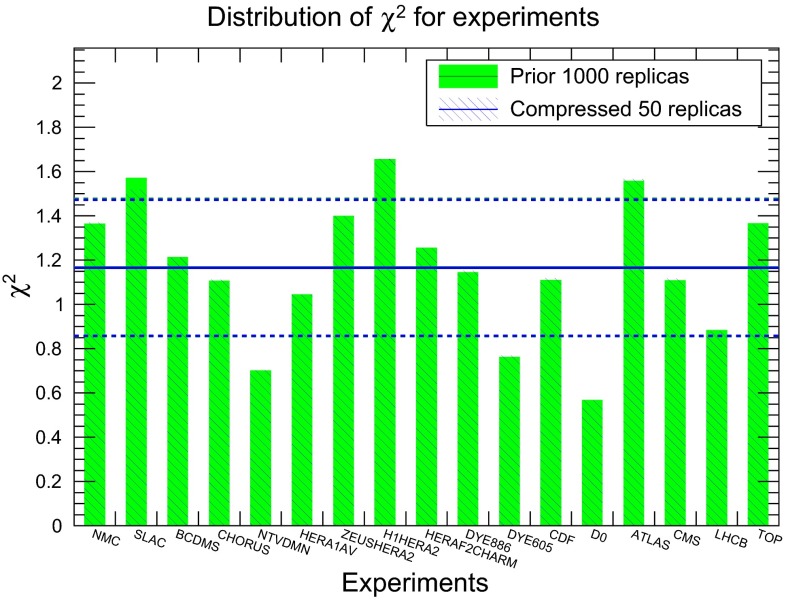
Distribution of $\chi ^2$ for all the experiments included in the NNPDF3.0 fit, comparing the original and the compressed PDF sets

First of all, we show the results for the compression of a native MC PDF set, for the case of the NNPDF3.0 NLO set with $\widetilde{N}_\mathrm{rep}=1000$ replicas. In Fig. 8 we compare the original and the compressed gluon and down quark at $Q^2=2$ GeV$^2$, using $N_\mathrm{rep}=50$ in the compressed set. Excellent agreement can be seen at the level of central values and variances. The comparison is also shown at a typical LHC scale of $Q=100$ GeV, finding similar agreement. The plots in this section have been obtained using the APFEL-Web online PDF plotter [58, 59]. The result that the central values of the original set are perfectly reproduced by the compressed set can also be seen from Fig. 9, where we show the distribution of $\chi ^2$ for all the experiments included in the NNPDF3.0 fit, comparing the original and the compressed PDF set, and find that they are indistinguishable.

**Fig. 10:**
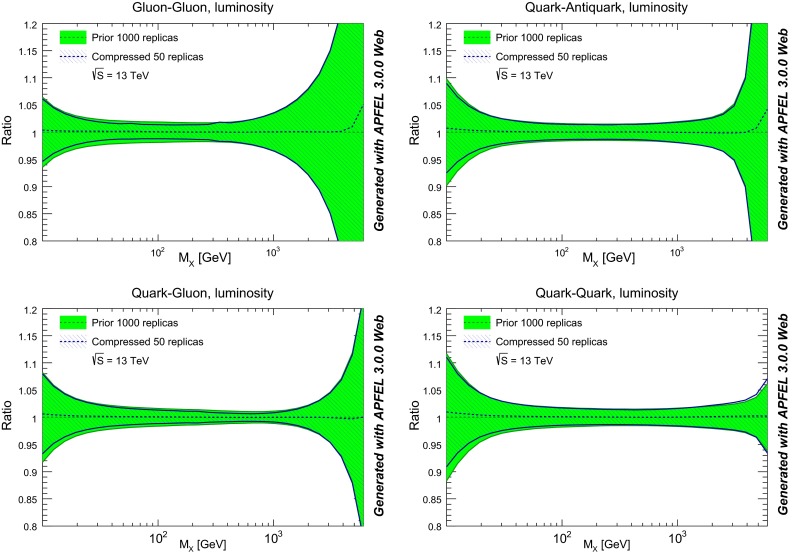
Comparison of PDF luminosities between the original and compressed NNPDF3.0 set, for the LHC 13 TeV as a function of the invariant mass of the final state $M_X$. From *top* to *bottom* and *left* to *right*, we show the gluon–gluon, quark–antiquark, quark–gluon, and quark–quark luminosities

Next, we compare in Fig. 10 the various PDF luminosities between the original and the compressed set at the LHC with center-of-mass energy of $\sqrt{s}=13$ TeV. We show the gluon–gluon, quark–antiquark, quark–gluon, and quark–quark luminosities. As in the case of the individual PDF flavors, good agreement is found in all the range of possible final state invariant masses $M_X$. Note that the agreement is also good in regions, like small $M_X$ and large $M_X$, where the underlying PDF distribution is known to be non-Gaussian.

**Fig. 11:**
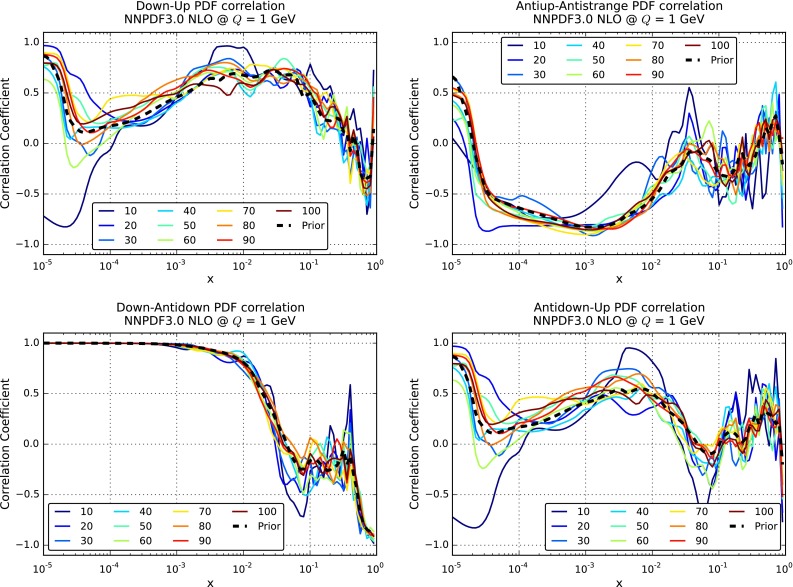
Comparison between the PDF correlations among different PDF flavors, as a function of Bjorken-*x*, for $Q=1$ GeV, for the original NNPDF3.0 set with $\widetilde{N}_\mathrm{rep}=1000$ replicas and the compressed sets for various values of $N_\mathrm{rep}$. From *top* to *bottom* and from *left* to *right*, we show the correlations between up and down quarks, between up and strange antiquarks, between down quarks and down antiquarks, and between up quarks and down antiquarks

It is also important to verify that not only central values and variances are reproduced, but also that higher moments and correlations are well reproduced by the compression. Indeed, one of the main advantages of the $N_\mathrm{rep}=1000$ replica sets of NNPDF as compared to the $N_\mathrm{rep}=100$ sets is that correlations should be reproduced more accurately in the former case. In Fig. 11 we show the results for the correlation coefficient between different PDFs, as a function of Bjorken-*x*, for $Q=1$ GeV. We compare the results of the original $\widetilde{N}_\mathrm{rep}=1000$ replica set, together with the results of the compressed sets for a number of $N_\mathrm{rep}$ values. From top to bottom and from left to right we show the correlations between up and down quarks, between up and strange antiquarks, between down quarks and down antiquarks, and between up quarks and down antiquarks. The correlations between PDF flavors have been computed using the suitable expression for Monte Carlo sets [31]. As we can see, correlations are reasonably well reproduced, already with $N_\mathrm{rep}=50$ the results of the compressed set and of the prior are very close to each other.

**Fig. 12:**
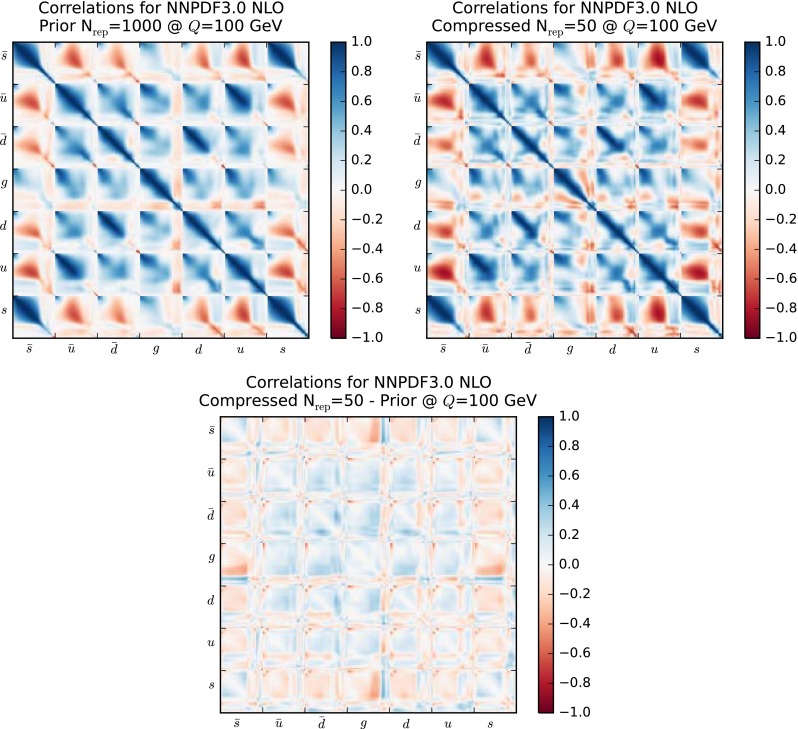
The correlation matrix of the NNPDF3.0 set with $\widetilde{N}_\mathrm{rep}=1000$ at $Q=100$ GeV. On the *right*, the same matrix for the NNPDF3.0 compressed set with $N_\mathrm{rep} = 50$ replicas. The *bottom plot* represents the difference between the two matrices. See text for more details

Another illustration of the fact that PDF correlations are maintained in the compression is provided by Fig. 12, where we show the correlation matrix of the NNPDF3.0 set at a scale of $Q=100$ GeV, comparing the prior with $\widetilde{N}_\mathrm{rep}=1000$ with the compressed set with $N_\mathrm{rep} = 50$ replicas. The correlation matrices presented here are defined in a grid of $N_x=50$ points in *x*, logarithmic distributed between $[10^{-5},1]$ for each flavor ($\bar{s},\bar{u},\bar{d},g,d,u,s$). To facilitate the comparison, in the bottom plot we show the differences between the correlation coefficients in the two cases. It is clear from this comparison that the agreement of the PDF correlations reported in Fig. 11 holds for the complete set of possible PDF combinations, in all the relevant range of Bjorken-*x*.

Having validated the compression results for a native MC set, we now turn to a discussion of the results of the compression for a combined MC PDF set.

### Compression of the CMC-PDFs

Now we turn to a similar validation study but this time for the CMC-PDFs. As we have discussed in Sect. 2, the combined MC set has been constructed by adding together $N_\mathrm{rep}=300$ replicas of NNPDF3.0, MMHT14, and CT14 each, for a total of $\widetilde{N}_\mathrm{rep}=900$ replicas. Starting from this prior set, the compression algorithm has been applied as discussed in Sect. 3, and we have produced CMC-PDF sets for a number of values of $N_\mathrm{rep}$ from 5 to 250 replicas, using the settings from Sect. 3.2.5.

**Fig. 13:**
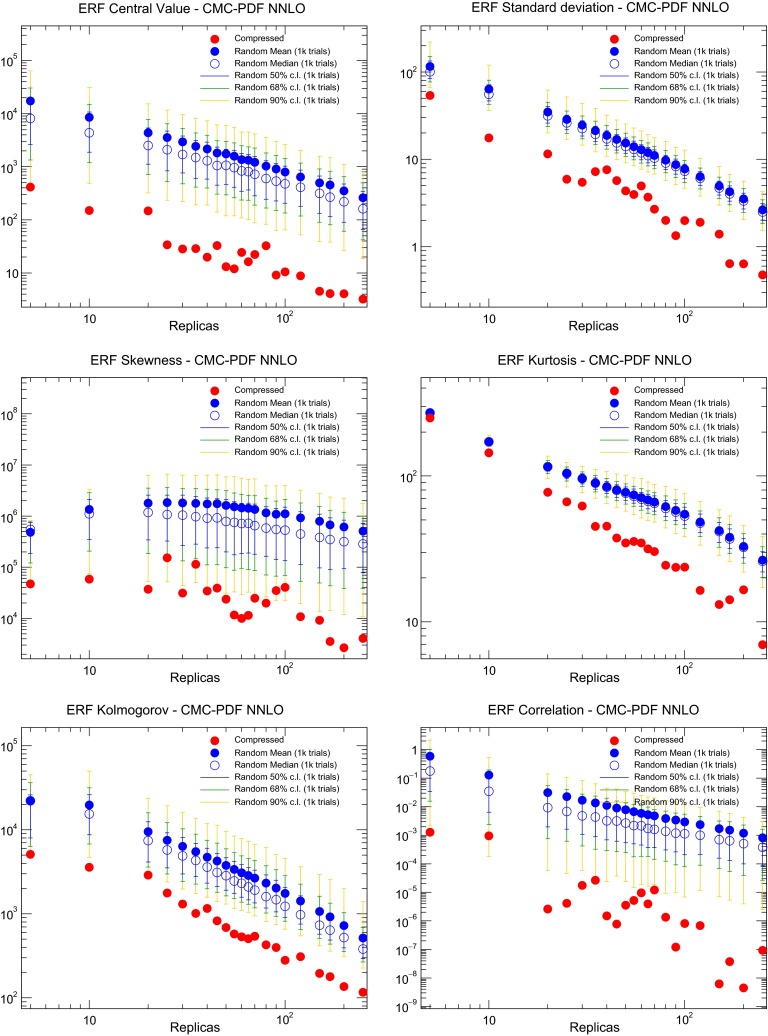
Same as Fig. 7 for the CMC-PDFs, starting from the prior with $\widetilde{N}_\mathrm{rep}=900$ replicas

We have verified that the performance of the compression algorithm is similar regardless of the prior. To illustrate this point, in Fig. 13 we show the corresponding version of Fig. 7, namely the various contributions to the error function, for the case of compression of the CMC-PDF sets. We see that also in the case of the CMC-PDF sets the compression improves the ERF as compared to random selections by an order of magnitude or even more.

**Fig. 14:**
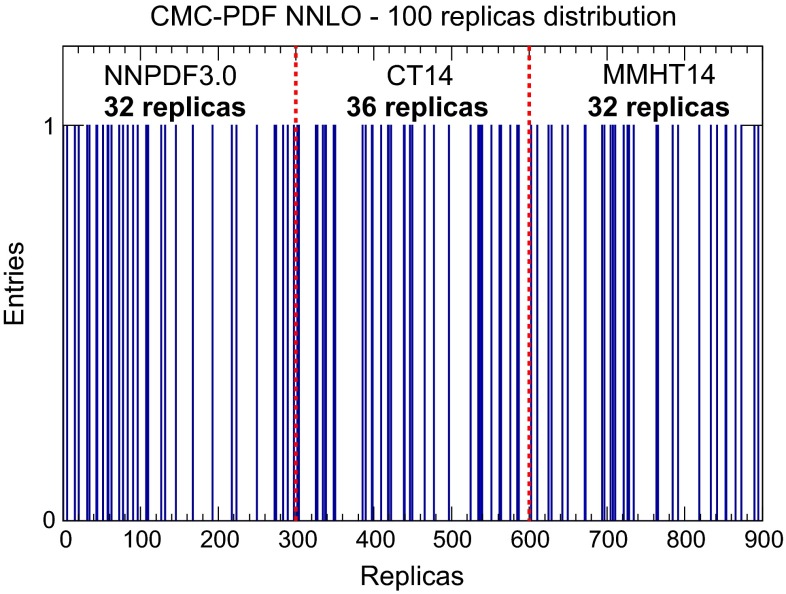
Replicas of the original combined set of $\widetilde{N}_\mathrm{rep}=900$ replicas selected for the compression with ${N}_\mathrm{rep}=100$ replicas, classified for each of the three input PDF sets

It is interesting to determine, for a given compression, how many replicas are selected from each of the three PDF sets that enter the combination. Given that originally we assign equal weight to the three sets, that is, the same number of replicas, we expect that if the compression algorithm is unbiased the number of replicas from each set after the compression should also be approximately the same. We have verified that this is indeed the case, for instance, in Fig. 14 we show, for a compression with $N_\mathrm{rep}=100$ replicas, how the replicas of the original distribution are selected: we see that a similar number has been selected from NNPDF3.0, CT14, and MMHT14: 32, 36, and 32 replicas, respectively, in agreement with our expectations.

**Fig. 15:**
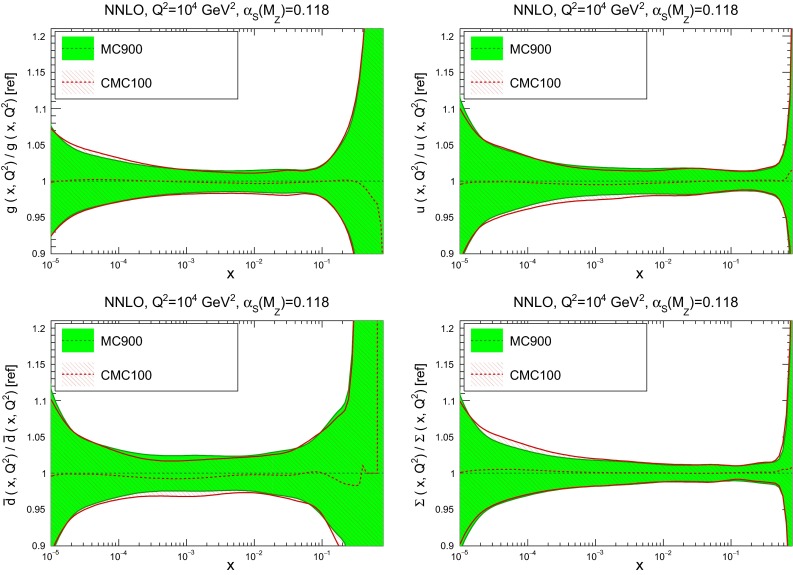
Comparison of the PDFs between the original Monte Carlo combination of NNPDF3.0, CT14, and MMHT14, MC900, with the compressed CMC100 PDFs. We show the gluon, up quark, down antiquark, and total quark singlet, as ratios to the prior for $Q^2=10^4$ GeV$^2$

We now address the comparison between the MC900 prior and the new CMC-PDFs. For illustration, we will show results for $N_\mathrm{rep}=100$, with the understanding that using a larger number of replicas would improve even further the agreement with the prior. In Fig. 15 we show the comparison of the PDFs between the original Monte Carlo combination of NNPDF3.0, CT14 and MMHT14, with $\widetilde{N}_\mathrm{rep}=900$ replicas, with the corresponding compressed set with $N_\mathrm{rep}=100$ replicas. We show the gluon, up quark, down antiquark, and strange quark, as ratios to the prior set at a typical LHC scale of $Q=100$ GeV. We see that in all cases the agreement is sufficiently good.

**Fig. 16:**
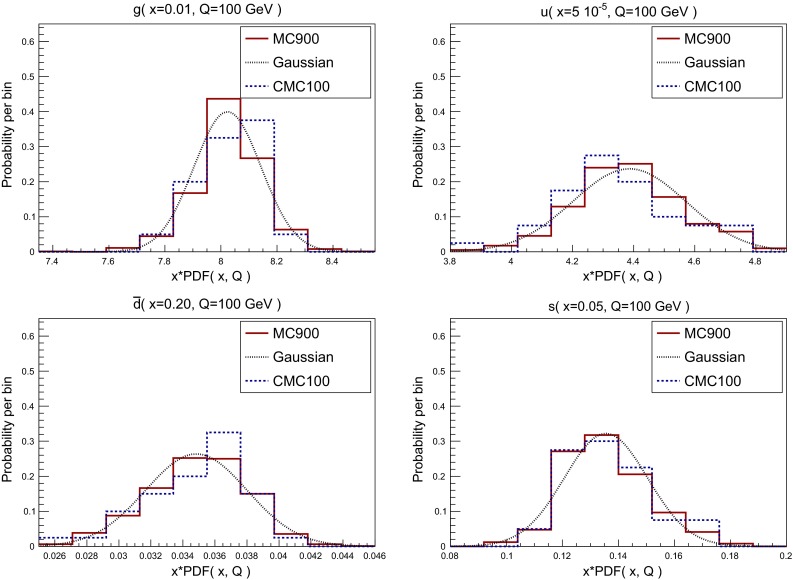
Same as Fig. 2 now with the comparison between MC900 and CMC100. The Gaussian curve has the same mean and variance as the MC900 prior

In Fig. 16 we show the same as in Fig. 2, namely the histograms representing the distribution of the values of the PDFs over the Monte Carlo replicas for different flavors and values of (*x*, *Q*), now comparing the original and compressed CMC-PDFs with $\widetilde{N}_\mathrm{rep}=900$ and $N_\mathrm{rep}=100$, respectively. As was done in Figs. 2 and 16 we also show a Gaussian with mean and variance determined from the prior $\widetilde{N}_\mathrm{rep}=900$ CMC-PDF.

**Fig. 17:**
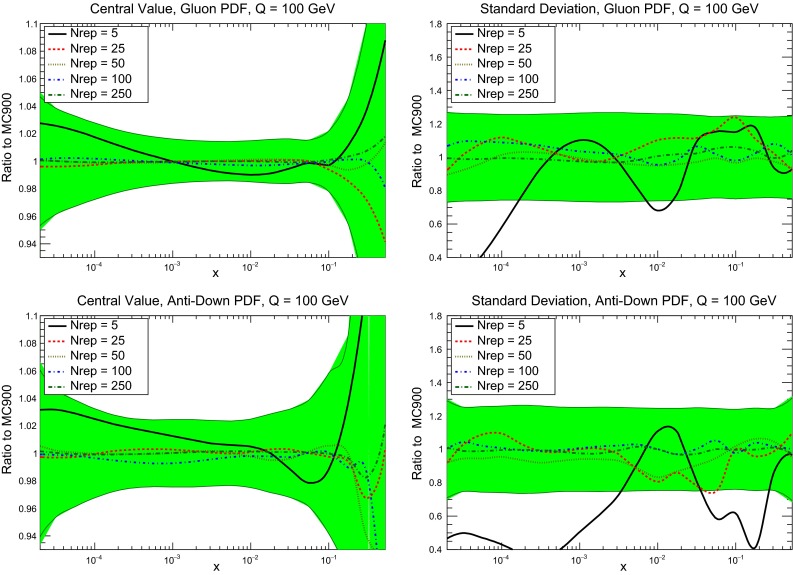
Comparison of the central values (*left plots*) and one-sigma intervals (*right plots*) for the CMC-PDFs with different values of $N_\mathrm{rep}$ (5, 25, 50, 100, and 250, respectively), for the gluon (*upper plots*) and the down antiquark (*lower plots*). Results are shown normalized to the central value and the standard deviation of the MC900 prior combined set, respectively. We also show the one-sigma PDF band (*left plots*) and the variance of the variance (*right plots*) as a full *green* band

To gauge the dependence of the agreement between the prior and the compressed Monte Carlo sets, it is illustrative to compare central values and variances for the different values of $N_\mathrm{rep}$ in the compression. This comparison is shown for the gluon and the down antiquark in Fig. 17. In the left plots, we compare the central value of the PDF for different values of $N_\mathrm{rep}$, normalized to the prior result. We also show the one-sigma PDF band, which is useful to compare the deviations found in the compressed set with the typical statistical fluctuations. We see that starting from $N_\mathrm{rep}\simeq 25$ replicas, the central values of the compressed sets fluctuate much less than the size of the PDF uncertainties. In the right plot of Fig. 17 we show the corresponding comparison at the level of standard deviations, again normalized to the standard deviation of the prior set. Here for reference the green band shows the variance of the variance itself, which is typically of the order of 20–30 % in a Monte Carlo PDF set [31]. Here we see that with $N_\mathrm{rep}\simeq 100$ replicas or more, the variance of the compressed set varies by a few percent at most, much less than the statistical fluctuations of the PDF uncertainty itself.

**Fig. 18:**
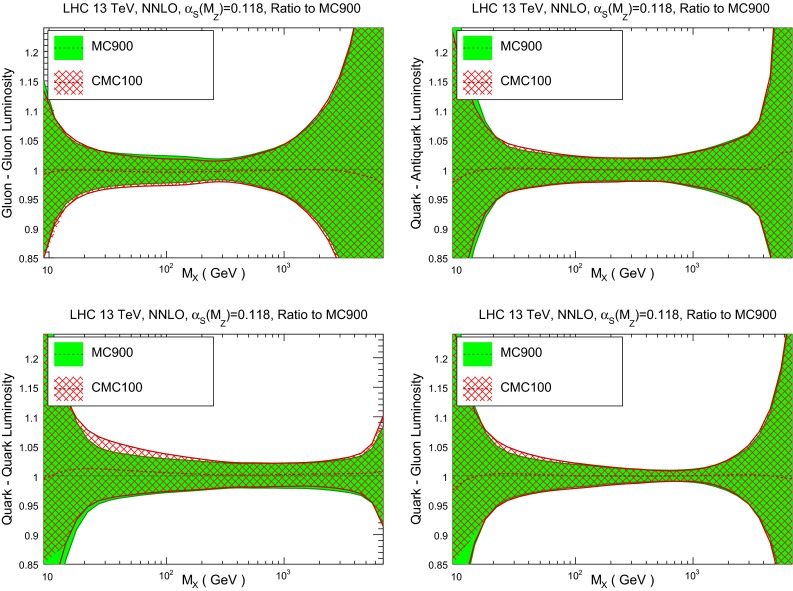
Same as Fig. 10 for the comparison between the prior set MC900 and the compressed set CMC100

As in the case of the native Monte Carlo sets, it is also useful here for the CMC-PDFs to compare the parton luminosities between the original and the compressed sets. This comparison is shown in Fig. 18, which is the analog of Fig. 10 in the case of CMC-PDFs. As in the case of the native sets, we find also here good agreement at the level of PDF luminosities. As we will see in the next section, this agreement will also translate to all LHC cross sections and differential distributions that we have explored.

**Fig. 19:**
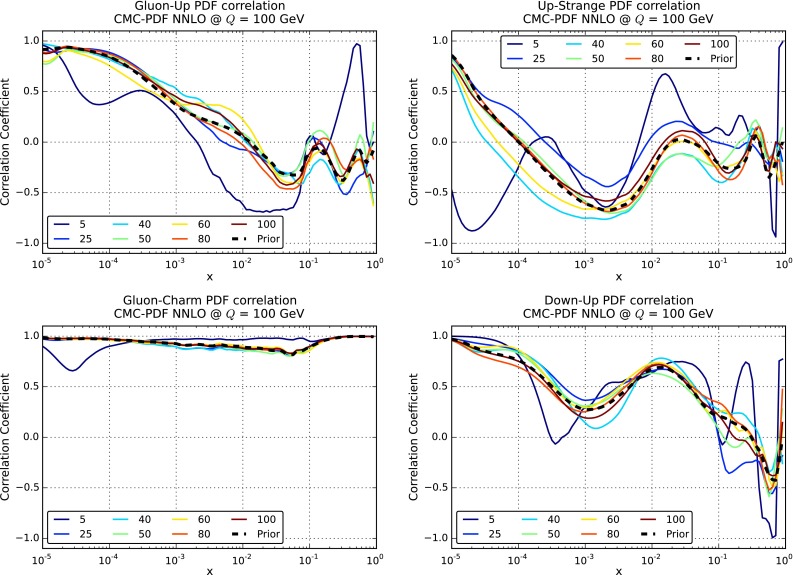
Same as Fig. 11 for correlation coefficients of the CMC-PDFs, evaluated at $Q=100$ GeV, for a range of values of $N_\mathrm{rep}$ in the compressed set, from 5 to 100 replicas, compared with the prior MC900 result. From *left* to *right* and from *top* to *bottom* we show the correlation between gluon and up quark, between up and strange quarks, between gluon and charm quark, and between the down and up quarks

Having verified in a number of ways that central values and variances of the PDFs are successfully preserved by the compression, we turn to a study of the PDF correlations. We have verified that a similar level of agreement as in the case of the native MC sets, Fig. 11, is achieved also here. To illustrate this point, in Fig. 19 we show a comparison of the correlation coefficients as a function of *x*, for $Q=100$ GeV, for different PDF combinations, between the original CMC-PDF set with $\widetilde{N}_\mathrm{rep}=900$ replicas and the compressed sets for different values of $N_\mathrm{rep}$. From left to right and from top to bottom we show the correlation between gluon and up quark, between up and strange quarks, between gluon and charm quark, and between the down and up quarks. We see that already with $N_\mathrm{rep}=100$ replicas the result for the correlation is close enough to the prior with $\widetilde{N}_\mathrm{rep}=900$ replicas.

**Fig. 20:**
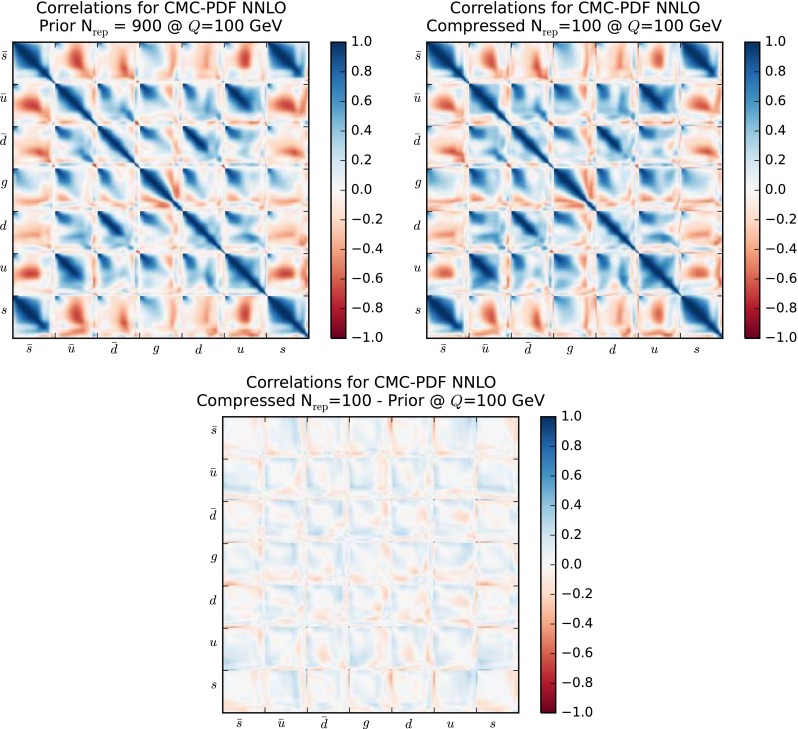
Same as Fig. 12 for the correlation matrix of the CMC-PDFs at $Q=100$ GeV, comparing the prior combination MC900 (*left plot*) and the CMC-PDF100 set (*right plot*). In the *bottom plot* we show the difference between the correlation coefficients in the two cases

The analogous version of Fig. 12 for the correlation matrix of the CMC-PDFs is shown in Fig. 20. As in the case of the native MC sets, also for the CMC-PDFs the broad pattern of the correlation matrix of the original combination with $\widetilde{N}_\mathrm{rep}=900$ replicas is maintained by the compression to $N_\mathrm{rep}=100$ replicas, as is quantified by the bottom plot, representing the differences between the correlation coefficients in the two cases.

## CMC-PDFs and LHC phenomenology

Now we present the validation of the compression algorithm applied to the combination of Monte Carlo PDF sets for a variety of LHC cross sections. We will compare the results of the original combined Monte Carlo set MC900 with those of the CMC-PDFs with $N_\mathrm{rep}=100$ replicas (CMC-PDF100). This validation has been performed both at the level of inclusive cross sections and of differential distributions with realistic kinematical cuts. All cross sections will be computed with the NNLO sets, even when the hard cross sections are computed at NLO, which is suitable for the present illustration purposes.

First of all, we compare the MC900 prior and the CMC-PDFs for benchmark inclusive LHC cross sections, and then we perform the validation for LHC differential distributions including realistic kinematical cuts. In the latter case we use fast NLO interfaces for the calculation of these LHC observables: this allows us to straightforwardly repeat the validation when different PDF sets are used for the compression without the need to repeat any calculation. Finally, we verify that the correlations between physical observables are also maintained by the compression algorithm, both for inclusive cross sections and for differential distributions.

### LHC cross sections and differential distributions

**Fig. 21:**
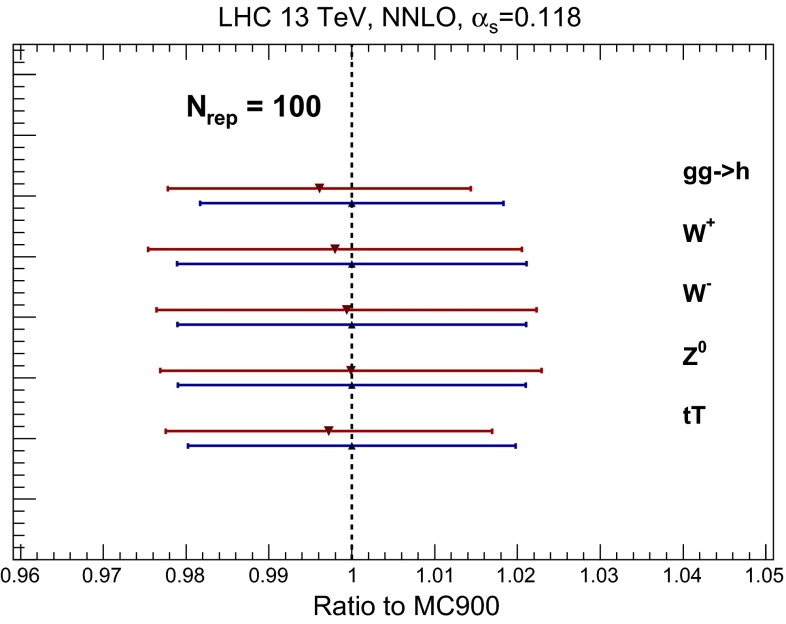
Comparison of the predictions of the Monte Carlo combined prior MC900 with those of the CMC-PDFs with $N_\mathrm{rep}=100$ replicas, normalized to the central value of the former, for a number of benchmark inclusive NNLO cross sections at the LHC with $\sqrt{s}=13$ TeV. The *error bands* correspond to the PDF uncertainty bands for each of the sets. See text for more details

We begin with the validation of the CMC-PDF predictions at the level of inclusive cross sections. The following results have been computed for the LHC at a centre-of-mass energy of 13 TeV. In Fig. 21 we compare the results obtained with the prior Monte Carlo combined set and with the CMC-PDFs with $N_\mathrm{rep}=100$ replicas, everything normalized to the central value of the prior set. The processes that have been included in Fig. 21 are the same as those considered in the benchmark comparisons of Sect. 2.2. As we can see from Fig. 21, in all cases the agreement at the central-value level is always at the permille level, and also the size of the PDF uncertainties is very similar between the original and compressed set. Taking into account the fluctuations of the PDF uncertainty itself, shown in Fig. 17, it is clear that the predictions from the original and the compressed sets are statistically equivalent.

Having established that the compression works for total cross sections, one might question if perhaps the accuracy degrades when we move to differential distributions, especially if one considers extreme regions of the phase space and the effects of realistic final state kinematical cuts. To verify that this is not the case, now we consider a number of differential processes computed using MCFM [60] and NLOjet++ [61] interfaced to APPLgrid [62] as well as MadGraph5_aMC@NLO [37] interfaced to aMCfast [63] and APPLgrid. All processes are computed for $\sqrt{s}=7$ TeV, and the matrix-element calculations have been performed at fixed NLO perturbative order. The advantage of using fast NLO grids is that it is straightforward to repeat the validation without having to redo the full NLO computation when a different set of input PDFs is used for the combination. Note that while for simplicity we only show the results for selected bins, we have verified that the agreement also holds for the complete differential distribution.

**Fig. 22:**
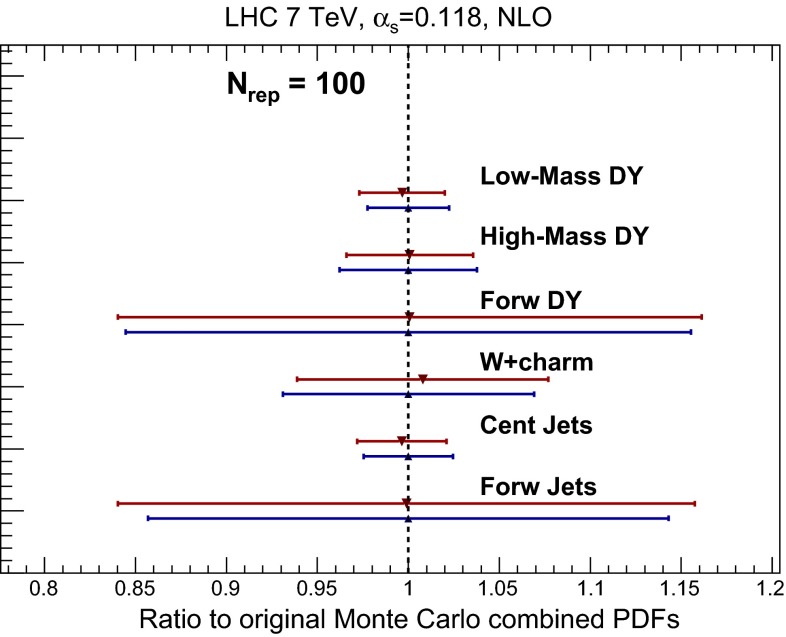
Same as Fig. 21, for a variety of NLO differential distributions computed with MCFM and NLOjet++ interfaced to APPLgrid for the LHC with $\sqrt{s}=7$ TeV. See text for the details of the choice of binning in each process

The corresponding version of Fig. 21 for the case of LHC 7 TeV differential distributions is shown in Fig. 22. The theoretical calculations are provided for the following processes:

- The ATLAS high-mass Drell–Yan measurement [64], integrated over rapidity $|y_{ll}|\le 2.1$, and binned as a function of the di-lepton invariant mass pair $M_{ll}$. Here we show the prediction for the highest mass bin, $M_{ll}\in \left[ 1.0,1.5\right]$ TeV.
- The CMS double differential Drell–Yan measurement [65] in the low-mass region, $20~\mathrm{GeV} \le M_{ll} \le 30$ GeV, as a function of the di-lepton rapidity $y_{ll}$. The prediction is shown for the lowest rapidity bin, $y_{ll}\in \left[ 0.0,0.1\right]$.
- The CMS $W^+$ lepton rapidity distribution [66]. The prediction is shown for the lowest rapidity bin, $y_{l}\in \left[ 0.0,0.1\right]$.
- The CMS measurement of $W^+$ production in association with charm quarks [67], as a function of the lepton rapidity $y_l$. The prediction is shown for the lowest rapidity bin, $y_{l}\in \left[ 0.0,0.3\right]$.
- The ATLAS inclusive jet production measurement [68] in the central rapidity region, $|y_\mathrm{jet}|\le 0.3$, as a function of the jet $p_T$. The prediction is shown for the lowest $p_T$ bin, $p_{T}\in \left[ 20,30\right]$ GeV.
- The same ATLAS inclusive jet production measurement [68] now in the forward rapidity region, $3.6 \le |y_\mathrm{jet}|\le 4.4$, as a function of the jet $p_T$. The prediction is shown for the highest $p_T$ bin, $p_{T}\in \left[ 110,160\right]$ GeV.

More details as regards the selection cuts applied to these processes can be found in the original references and in the NNPDF3.0 paper [16], though note that here no comparison with experimental data is attempted. The various observables of Fig. 22 probe a wide range of PDF combinations, from light quarks and antiquarks (low- and high-mass Drell–Yan) and strangeness (*W*+charm) to the gluon (central and forward jets) in a wide range of Bjorken-*x* and momentum transfers $Q^2$.

As we can see from Fig. 22, the level of the agreement between the MC900 prior and the CMC-PDFs with $N_\mathrm{rep}=100$ is similar to that of the inclusive cross sections.

This is also true for other related processes that we have also studied, but that are not shown explicitly here. This agreement is of course understood from the fact that the compression is performed at the level of parton distributions, as shown in Sect. 4. Note also that the agreement found for the processes in Fig. 22 is particularly remarkable since in some cases, like forward Drell–Yan or forward jet production, the underlying PDFs are probed at large-*x*, where deviations from the Gaussian behavior are sizable: even in this case, the compression algorithm is successful in reproducing the mean and variance of the prior probability distribution.

Another illustrative way of checking that the compression algorithm really preserves the non-Gaussian features of the prior is provided by the probability distribution of specific LHC cross sections in which such features are clearly observed. To better visualize the probability density $P(\sigma )$ estimated from the Monte Carlo sample we use the *Kernel Density Estimation* (KDE) method. In this technique, the probability distribution is obtained by averaging a kernel function *K* centered at the predictions $\{\sigma _{i}\}$ obtained for each individual PDF replica:

22 $$\begin{aligned} P(\sigma )={{1}\over {N_{\mathrm{rep}}}}\sum _{i=1}^{N_{\mathrm{rep}}}K\left( \sigma -\sigma _{i}\right) . \end{aligned}$$

Here we choose the function *K* to be a normal distribution, that is,

23 $$\begin{aligned} K(\sigma -\sigma _{i})={{1}\over {h\sqrt{2\pi }}}e^{{{-(\sigma -\sigma _{i})^{2}}\over {h}}}, \end{aligned}$$

where we set the parameter *h*, known as bandwidth, so that it is the optimal choice if the underlying data was Gaussian. This choice is known as the Silverman rule.^2^

**Fig. 23:**
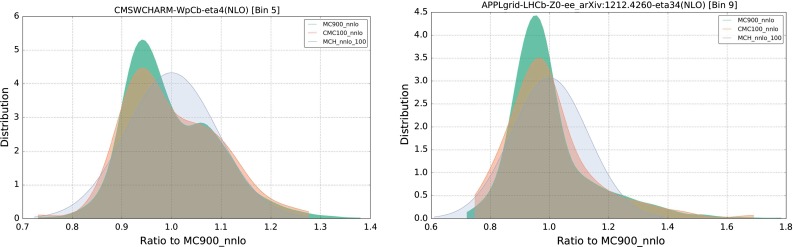
The probability distribution for two LHC cross sections: the CMS *W*+charm production in the most forward bin (*left plot*) and the LHCb $Z\rightarrow e^+e^-$ rapidity distribution for $\eta _{Z}= 4$ (*right plot*). We compare the original prior MC900 with the results from the CMC-PDF100 and MCH100 reduced sets

In Fig. 23 we compare the probability distributions, obtained using the KDE method, for two LHC cross sections: the CMS *W*+charm production in the most forward bin (left plot) and the LHCb $Z\rightarrow e^+e^-$ rapidity distribution for $\eta _{Z}= 4$ (right plot). We compare the original prior MC900 with the CMC-PDF100 and MCH100 reduced sets. In the case of the *W*+charm cross section, which is directly sensitive to the poorly known strange PDF, the prior shows a double-hump structure, which is reasonably well reproduced by the CMC-PDF100 set, but that disappears if a Gaussian reduction, in this case MCH100, is used. For the LHCb forward *Z* production, both the prior and CMC-PDF100 are significantly skewed, a feature which is lost in the Gaussian reduction of MCH100.

### Correlations between LHC cross sections

Any reasonable algorithm for the combination of PDF sets should reproduce not only the central values and the variances of the prior distribution, but also the correlations between physical observables. This is relevant for phenomenological applications at the LHC, where PDF-induced correlations are used for instance to determine the degree of correlation of the systematic uncertainties between different processes. Using the PDF4LHC recommendations, the PDF-induced correlations between different Higgs production channels were estimated in Ref. [55], and this information is now extensively used in the Higgs analyses of ATLAS and CMS.

**Fig. 24:**
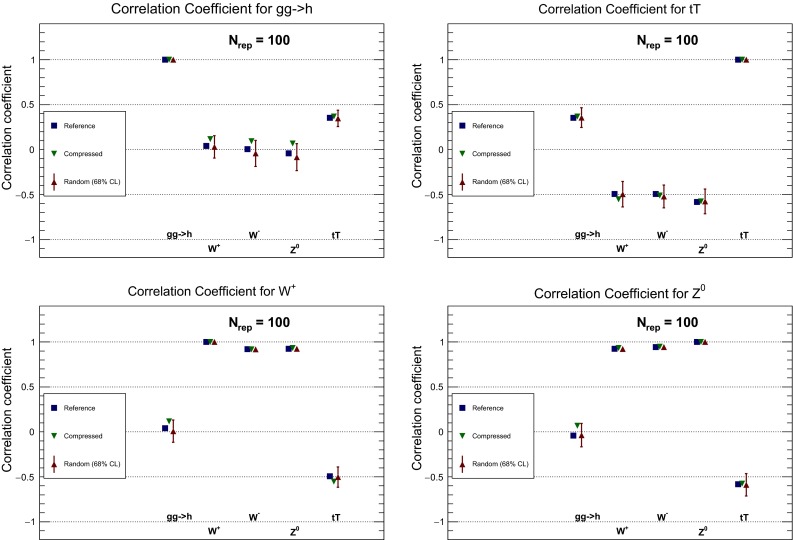
Comparison of the correlation coefficients computed from the reference Monte Carlo combined set and from the CMC-PDFs with $N_\mathrm{rep}=100$ replicas. We show here the results for the correlations between the inclusive LHC cross sections, using the settings described in the text. Each plot contains the correlation coefficient of a given cross section with respect to all the other inclusive cross sections considered here. To gauge the effectiveness of the compression algorithm, we also show the 68 % confidence-level interval for the correlation coefficients computed from $N_\mathrm{rand}=1000$ random partitions of $N_\mathrm{rep}=100$ replicas each

**Fig. 25:**
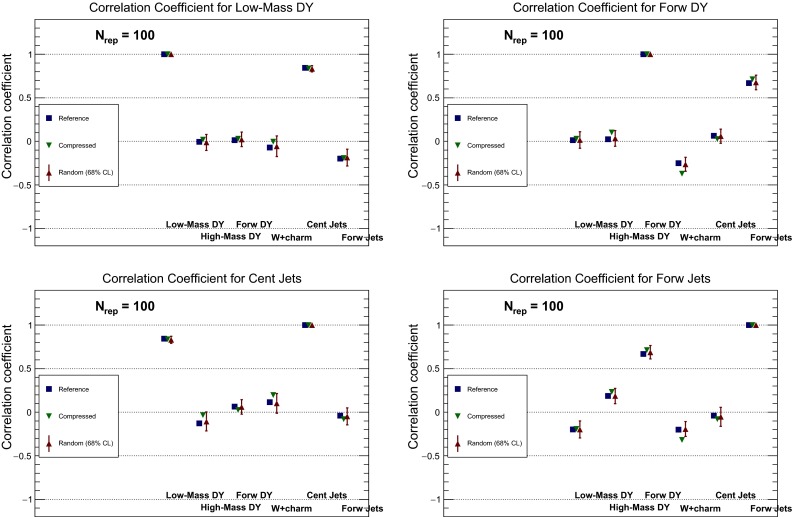
Same as Fig. 24 for some of the various LHC NLO differential cross sections discussed in the text. From *top* to *bottom* and from *left* to *right* we show the correlations for low-mass Drell–Yan, forward Drell–Yan, and central and forward jets

To validate that the compression algorithm presented here also maintains the correlations of the original set, we have computed the correlations between all processes used in the previous section, both for the MC900 prior and for the CMC-PDF100 set. The results are shown in Fig. 24, for the NLO and NNLO inclusive cross sections shown in Figs. 21 and 25, for the case of differential distributions shown in Fig. 22. We have also verified that from $N_\mathrm{rep}\simeq 50$ replicas onwards the correlations are very well reproduced by the compressed set.

To gauge the effectiveness of the compression algorithm, in Figs. 24 and 25 we also show the 68 % confidence-level interval for the correlation coefficients computed from $N_\mathrm{rand}=1000$ random partitions of $N_\mathrm{rep}=100$ replicas: we see the compression in general outperforms the results from a random selection of a $N_\mathrm{rep}=100$ replica set. The agreement of the correlations at the level of LHC observables is a direct consequence of course that correlations are maintained by the compression at the PDF level, as discussed in detail in Sect. 4.2. Only for very few cases the correlation coefficient of the CMC-PDF set is outside the 68 % confidence-level range of the random selections, and this happens only when correlations are very small to begin with, so this fact is not relevant for phenomenology.

To summarize, the results of this section show that at the level of LHC phenomenology, CMC-PDFs with $N_\mathrm{rep}=100$ replicas can be reliably used instead of the original Monte Carlo combination of PDF sets, thereby allowing a substantial reduction of the CPU-time burden associated with the calculation of the theory predictions for the original $\widetilde{N}_\mathrm{rep}=900$ replicas by almost a full order of magnitude.

## Summary and delivery

In this work we have presented a novel strategy for the combination of individual PDF sets, based on the Monte Carlo method followed by a compression algorithm. The resulting Compressed Monte Carlo PDFs, or CMC-PDFs for short, are suitable to be used to estimate PDF uncertainties in theoretical predictions of generic LHC processes. As compared to the original PDF4LHC recommendation, the new approach we advocate here is both more straightforward to use, based on a single combined PDF set, and less computationally expensive: $N_\mathrm{rep}\simeq 100$ replicas are enough to preserve the statistical features of the prior combination with sufficient accuracy for most relevant applications. Using as an illustration the combination of the recent NNPDF3.0, CT14, and MMHT14 NNLO sets, we have verified that the compression algorithm successfully reproduces the predictions of the prior combined MC set for a wide variety of LHC processes and their correlations.

The compressed PDF sets at NLO and NNLO, with $N_\mathrm{rep}=100$ replicas each, and $\alpha _s(M_Z)=0.118$, will be made available in LHAPDF6 [49] as part of the upcoming PDF4LHC 2015 recommendations. Additional members to estimate the combined PDF+$\alpha _s$ uncertainty will also be included in the same grid files, and new functions will be provided in LHAPDF 6.1.6 to facilitate the computation of this combined PDF+$\alpha _s$ uncertainty. In addition, we have also made publicly available the compression algorithm used in this work:

https://github.com/scarrazza/compressor

This compressor code [50] includes a script to combine Monte Carlo sets from different groups into a single MC set, the compression algorithm and the validation suite. A concise user manual for this code can be found in the appendix: the code produces CMC-PDF sets directly in the LHAPDF6 format ready to be used for phenomenological applications.

We would like to emphasize that it is beyond the scope of this paper to determine which specific PDF sets should be used in the present or future PDF4LHC combination: this is an issue in which only the PDF4LHC Steering Committee has the mandate to decide. We have used the most updated NNLO sets from NNPDF, CT, and MMHT for consistency with the current prescription, but using the publicly available code it is possible to construct CMC-PDFs from any other choice of sets. We note, however, that for the combination of PDF sets that are based on very different input datasets or theory assumptions as compared to the three global sets, the determination of the number of replicas from each set that should be included in the combination is a complex problem which is still to be understood.

Examples of applications where combined PDF sets, as implemented by the CMC-PDFs, should be used include the computation of PDF uncertainties for acceptances and efficiencies, due to extrapolations or interpolations, to estimate the PDF uncertainties in the extraction of Higgs couplings or other fundamental SM parameters such as $M_W$ from LHC data, and to obtain limits in searches for BSM physics. Even in these cases, whenever possible, providing results obtained using individual PDF sets should be encouraged, since such comparisons shed light on the origin of the total PDF uncertainties for each particular application, and provide guidance about how they might reduce this PDF uncertainty. Needless to say, in all PDF-sensitive Standard Model comparisons between experimental data and theory models, only the individual PDF sets should be used, rather than only a combined PDF set. The latter might be suitable only if PDF uncertainties are much smaller than all other theoretical and experimental uncertainties.

It is also important to emphasize that the CMC-PDFs, as well as any other method for the combination of PDF sets, do not replace the individual PDF sets: CMC-PDFs are simply a user-convenient method to easily obtain the results of the combination of the individual PDF sets. For this reason, it should be clear that whenever the CMC-PDF sets are used, not only the present publication should be cited, but also the original publications corresponding to the individual PDF sets used as input to the combination.

Let us conclude by stating the obvious fact that the availability of a method for the combination of different sets does not reduce, but if anything strengthens, the need to keep working in reducing the PDF uncertainties in the individual sets, both in terms of improved theory, more constraining data and refined methodology, as well as to continue the benchmarking exercises between groups that have been performed in the past [4, 69, 70] and that are instrumental to understand (and eventually reduce) the differences between different groups.

## A: The compression code

The numerical implementation of the compression algorithm used in this work is available in the compressor v1.0.0 package. Here we provide instructions about how to download, install, and run the code. The code was designed for Unix systems.

Download

Open a terminal and download the latest release available from

https://github.com/scarrazza/compressor/releases

or clone the master development branch from the GitHub repository:

Installation

Compressor requires three external public libraries in order to work properly: LHAPDF6^3^ [49], ROOT^4^ and GSL.^5^ In order to install the package compile with the configure script:  This operation will copy the bin/compressor binary to /usr/local/bin (or the location given by –prefix).

Running the code

After installing this package, the compressor program is available for the user. The first two arguments are required:

- REP: the number of required compressed replicas
- PDF prior name: the name of the prior LHAPDF6 grid
- energy$Q$: the input energy scale used by the compression algorithm (default = 1 GeV)
- seed: the random number seed (default = 0)
- compress: switches on/off the minimization step (default = true).

Output

After running compressor a folder with the prior set name is created.

The script /bin/compressor_buildgrid creates the compressed LHAPDF6 grid:

Finally, in order to generate the ERF plots place the /bin/compressor_validate.C script in the output folder and run:

## Footnotes

1 Note that Eq. (6.5) of Ref. [35] and the current LHAPDF 6.1.5 code contain a mistake which has been corrected in the Mercurial repository and the correction will be included in the upcoming LHAPDF 6.1.6 release; see Eq. (22) of Ref. [49].

2 It can be shown that this choice amounts to using a bandwidth of

24 $$\begin{aligned} h=\left( {{4s^{5}}\over {3N_{\mathrm{rep}}}}\right) ^{{{1}\over {5}}}, \end{aligned}$$

where *s* is the standard deviation of the sample.
